# Drug-Drug Interactions Between HIV Antivirals and Concomitant Drugs in HIV Patients: What We Know and What We Need to Know

**DOI:** 10.3390/pharmaceutics17010031

**Published:** 2024-12-28

**Authors:** Emanuela De Bellis, Danilo Donnarumma, Adele Zarrella, Salvatore Maria Mazzeo, Annarita Pagano, Valentina Manzo, Ines Mazza, Francesco Sabbatino, Graziamaria Corbi, Pasquale Pagliano, Amelia Filippelli, Valeria Conti

**Affiliations:** 1 School “Clinical and Translational Oncology (CTO)”, Scuola Superiore Meridionale, University of Naples “Federico II”, 80138 Naples, Italy; emanuela.debellis@unina.it (E.D.B.); d.donnarumma@ssmeridionale.it (D.D.); 2 Postgraduate School of Clinical Pharmacology and Toxicology, University of Salerno, 84081 Baronissi, Italy; azarrella@unisa.it (A.Z.); s.mazzeo9@studenti.unisa.it (S.M.M.); annpagano@unisa.it (A.P.); imazza@unisa.it (I.M.); 3 Clinical Pharmacology Unit, San Giovanni di Dio e Ruggi d’Aragona University Hospital, 84131 Salerno, Italy; vmanzo@unisa.it (V.M.); afilippelli@unisa.it (A.F.); 4 Department of Medicine, Surgery, and Dentistry, Scuola Medica Salernitana, University of Salerno, 84081 Baronissi, Italy; fsabbatino@unisa.it (F.S.); ppagliano@unisa.it (P.P.); 5 Oncology Unit, University Hospital “San Giovanni di Dio e Ruggi d’Aragona”, 84131 Salerno, Italy; 6 Department of Translational Medical Sciences, University of Naples “Federico II”, 80131 Naples, Italy; graziamaria.corbi@unina.it; 7 Infectious Diseases Unit, San Giovanni di Dio e Ruggi d’Aragona University Hospital, 84131 Salerno, Italy

**Keywords:** HIV, people living with HIV, HAART, drug-drug interactions, pharmacokinetics, adverse drug events, confection, polypharmacotherapy, comorbidities, protease inhibitors, integrase inhibitors

## Abstract

Highly active antiretroviral therapy has led to a significant increase in the life expectancy of people living with HIV. The trade-off is that HIV-infected patients often suffer from comorbidities that require additional treatment, increasing the risk of Drug-Drug Interactions (DDIs), the clinical relevance of which has often not been determined during registration trials of the drugs involved. Therefore, it is important to identify potential clinically relevant DDIs in order to establish the most appropriate therapeutic approaches. This review aims to summarize and analyze data from studies published over the last two decades on DDI-related adverse clinical outcomes involving anti-HIV drugs and those used to treat comorbidities. Several studies have examined the pharmacokinetics and tolerability of different drug combinations. Protease inhibitors, followed by nonnucleoside reverse transcriptase inhibitors and integrase inhibitors have been recognized as the main players in DDIs with antivirals used to control co-infection, such as Hepatitis C virus, or with drugs commonly used to treat HIV comorbidities, such as lipid-lowering agents, proton pump inhibitors and anticancer drugs. However, the studies do not seem to be consistent with regard to sample size and follow-up, the drugs involved, or the results obtained. It should be noted that most of the available studies were conducted in healthy volunteers without being replicated in patients. This hampered the assessment of the clinical burden of DDIs and, consequently, the optimal pharmacological management of people living with HIV.

## 1. Introduction

Highly Active Antiretroviral Therapy (HAART) for the treatment of patients with *Human Immunodeficiency Virus* (*HIV*) has transformed this infection from a fatal to a manageable chronic condition [1].

In fact, the number of deaths from Acquired Immunodeficiency Syndrome (AIDS), which develops in inadequately treated HIV+ patients, is decreasing worldwide and the life expectancy of People Living With HIV (PLWH) treated with HAART increases from 10 to 25 years when compared with patients who do not receive this therapy [1,2].

HAART is based on the combination of different antiretroviral agents, divided into six main classes: Nucleoside/Nucleotide Reverse Transcriptase Inhibitors (NRTIs), Non-nucleoside Reverse Transcriptase Inhibitors (NNRTIs), Protease inhibitors (PIs), Integrase Inhibitors (INIs), Fusion inhibitors (FIs), Chemokine Receptor Antagonists (CCR5 Antagonists) [3]. These drugs act at different stages of the viral life cycle and have different molecular targets. NRTIs/NNRTIs, PIs, and INIs inhibit, respectively, proteins p66/p51, p11, and p32 encoded by the *pol* gene; FIs act by binding glycoprotein 120 and 41 encoded by the *env* gene; CCR5 Antagonist acts by binding the human trans-membrane receptor of chemokine CCR5, preventing the virus from attaching to CD4^+^ T lymphocytes.

HAART reduces viremia and increases the number of CD4^+^ T lymphocytes, thereby restoring the patient’s immune activity [3,4]. In HIV+ patients, assessment of viral load and CD4^+^ T lymphocyte count is performed periodically to monitor the efficacy and tolerability of treatment [5,6]. HIV+ patients may develop comorbidities [7], resulting in the need for polypharmacotherapy, which in turn exposes them to an increased risk of adverse drug events (ADEs), including those related to drug-drug interactions (DDIs).

The term ‘DDI’ refers to the phenomenon whereby the pharmacodynamic (PD) or pharmacokinetic (PK) profiles of a drug are altered by the concomitant administration of other pharmacologically active agents, potentially causing ADEs. For instance, the concomitant use of multiple drugs may cause toxic synergism or induce or inhibit transporters such as P-glycoprotein (P-gp) or phase I (e.g., cytochrome P450 (CYP) isoforms) or phase II (e.g., uridine 5′-diphosphate-glucuronosyltransferase 1A1, UGT1A1) metabolic enzymes.

In particular, the use of PK boosters, such as ritonavir (RTV) and cobicistat, which act as inhibitors of various cytochrome (CYP) P450 enzyme isoforms, may influence the CYP450-dependent metabolism of co-administered drugs, thereby increasing their plasma exposure [8,9,10]. One of the most important goals achieved with the introduction of HAART is the treatment of PLWH with concomitant infections such as malaria, tuberculosis, and Hepatitis C Virus (HCV). Coinfections can affect the therapeutic outcomes of patients per se, and drugs used to treat co-infected patients can interact with anti-HIV agents. This adds to the inevitable occurrence of *HIV* mutations associated with drug resistance and further complicates the long-term management of patients [11,12]. DDI-related ADEs can decrease adherence and persistence to therapy, accelerating the onset of drug resistance and leading to the need for additional drug therapies. In clinical practice, this leads to an increase in specialist visits and hospitalizations, with a negative economic impact [13,14].

When polypharmacotherapy is administered chronically in all medical areas, special attention should be paid to treatment monitoring, establishing a link between all clinicians involved in patient care, including general practioners [15,16]. However, data on the mechanisms and real impact in clinical practice associated with the concomitant use of multiple drugs are scarce and often inconclusive.

This review aims to summarize and analyze data from studies, published between 2000 and 2024, on DDIs and DDI-related ADEs involving drugs belonging to HAART and those for the treatment of comorbidities, with a focus on drugs used in patients with HIV and coinfections.

## 2. Studies Investigating DDIs Between HAART and Co-Administered Drugs in Healthy Subjects

Seventy-six studies, enrolling a total of 2044 healthy subjects, were analyzed (Table 1). Among them, 57 were Randomized Clinical Trials (RCTs) [17,18,19,20,21,22,23,24,25,26,27,28,29,30,31,32,33,34,35,36,37,38,39,40,41,42,43,44,45,46,47,48,49,50,51,52,53,54,55,56,57,58,59,60,61,62,63,64,65,66,67,68,69,70,71,72,73], 5 were crossover Clinical Trials (CTs) [74,75,76,77,78], 9 were referred to as PK studies [79,80,81,82,83,84,85,86,87], and 5 were DDIs studies [88,89,90,91,92]. Regardless of the study design, the main aim was to evaluate whether DDIs lead to changes in PK and, consequently, in the drug safety and efficacy profile.

The drugs involved were antivirals belonging to the HAART regimen, such as PIs, INIs, and NNRTIs and drugs used to treat comorbidities and coinfections such as Hepatitis B Virus (HBV) and HCV, malaria, and tuberculosis (TB). In addition, several studies report data on contraceptives, the proton pump inhibitor (PPI) omeprazole (OME), and antiacids, antidepressants, antiepileptics, statins, and supplements/food.

Among the INIs, that represent one of the first-line treatments for HIV infection [93], several studies have investigated possible changes in the PK of raltegravir (RAL) [18,20,21,28,29,45,66,67,69,74,86] or dolutegravir (DTG) [50,51,56,57,58,59,60,61,62,63,68,80,88] when these drugs were co-administered with other antivirals, antibiotics, contraceptives, anti-tuberculosis, anti-malarian, statins, and herb supplements. With regard to RAL, the studies overall did not find changes in the PK of the drug such as to require a change in dosing or other clinical interventions, nor DDIs potentially associated with adverse outcomes.

Only Iwamoto et al., who studied the possible interference between oral omeprazole (OME) 20 mg daily and a single oral dose RAL 400 mg in 28 healthy subjects, reported that this DDI caused an increase in the gastric pH and solubility of RAL resulting in a 3–4-fold increase in the Area Under Curve (AUC) and maximum plasma concentration (Cmax) of the antiviral and a consequent increase in its bioavailability [29].

Additional evidence is available on the possible involvement of DTG in clinically significant DDIs due to changes in PK parameters of DTG or co-administered drugs.

Brooks et al. highlighted possible ADEs mediated by cytokine release following the co-administration of this oral INI 50 mg daily with the oral isoniazid/rifapentine (INH/RPT) 900/900 mg daily regimen used as a treatment option in HIV+ patients with latent TB. The study was stopped because, after the third dose of INH-RPT, a severe flu-like syndrome associated with elevated aminotransferase levels occurred in 2 of the 4 enrolled subjects. In these subjects, the AUC of DTG was reduced by 46%, while that of INH increased by 67–92%. Despite the interruption of the study, this result appears very interesting because both increased aminotransferase and flu-like syndrome are ADEs recognised to be associated with the INH/RPT regimen [88].

Song et al. conducted several studies to assess changes in the activity profile of DTG or drugs/supplements co-administered with it [56,57,58,59,60,61,62,63,64]. As DTG is primarily metabolized by uridine 5′-diphospho-glucuronosyltransferase 1A1 (UGT1A1) and CYP3A4 [94], co-administration with potent inducers of these enzymes, such as carbamazepine (CBZ), can easily lead to changes in the PK profile of DTG. In fact, a statistically significant decrease in AUClast, Cmax, and Cτ of DTG was found to be 49, 33, and 73%, respectively. The authors suggested taking oral DTG 50 mg twice daily instead of once daily, similar to what is recommended when this antiviral is administered with other potent UGT1A1/CYP3A inducers such as tipranavir/ritonavir (TPV/r), efavirenz (EFV) and rifampicin [95,96].

Furthermore, it was shown that the AUCinf, Cmax and C24 of oral DTG 50 mg, were reduced by 39, 37 and 39%, respectively, when co-administered with fasting oral calcium carbonate 1200 mg and by 54, 57, and 56%, respectively, under similar conditions, when co-administered with oral iron fumarate 324 mg. For this reason, it is advisable to distance the intake of DTG two hours before or six hours after taking calcium and iron supplements [56].

The same authors evaluated whether food could alter the PK of DTG. Indeed, although without clinical consequences, the AUCinf increased by up to 66% when oral DTG was taken with high-fat foods [59]. Notably, accumulating evidence concerns the potential effect of DTG on the PK of metformin (MET). In particular, DTG, being a known inhibitor of organic cation transporter 2 (OCT2) [97], may increase the plasma concentration of drugs eliminated via OCT2, such as MET.

Song et al. showed that the AUClast and Cmax of MET increased by 79% and 66%, respectively, when this antidiabetic was administered with oral DTG 50 mg once daily and by 145% and 111%, respectively, with DTG 50 mg twice daily. For these reasons, when co-administering DTG, the dose of oral MET 500 mg twice daily should be monitored based on tight glycaemic control [63]. Ford et al. studied the influence of oral rifampin (RIF) 600 mg daily, an antibiotic commonly used to treat TB, on the PK of oral cabotegravir (CAB) 30 mg. RIF is an inducer of the CYP450 isoforms and UGT1A1, thus it is able to influence the metabolism of CAB. Therefore, co-administration of RIF/CAB is not recommended due to the possible significant decrease in plasma exposure to this antiretroviral agent [75]. It is noteworthy potential effects deriving from DDIs involving PIs in polypharmacy.

Fang et al. and Zhu et al. [24,73] evaluated the influence of the co-administration of OME with nelfinavir (NFV) and atazanavir/ritonavir (ATV/r), respectively.

The first study, although the precise mechanism has not yet been fully elucidated, showed that oral OME 40 mg daily is able to significantly reduce the AUClast, Cmax, and minimum plasma concentration (Cmin) of oral NFV 1250 mg twice daily (by 36, 37 and 39%, respectively) and even more so the PK parameters of the active NFV metabolite, M8 (by 92, 89 and 75%, respectively). For this reason, co-administration of these two drugs is not recommended, as it could lead to loss of viremic control [24].

The second study suggested that oral OME 20 mg had a less pronounced effect on the PK profile of oral ATV than the 40 mg dosage. Indeed, it is widely reported that co-administration of ATV/r 400/100 mg with OME 40 mg is strongly discouraged, because it results in a decrease in ATV bioavailability of up to 75% [98]. Specifically, co-administration of OME 20 mg with ATV/r 300/100 mg reduced the AUC and Cmin of ATV by 42% and 46%, respectively, while the Cmin of ATV was reduced by about 30% using ATV/r 400/100 mg. This highlights the importance of choosing the most appropriate dosage of ATV/r to anticipate the effects of this DDI.

Garraffo et al. [76] investigated the alterations potentially associated to TPV/r use in the PK profile of tadalafil (TAD), a phosphodiesterase 5 (PDE5) inhibitor widely used in the treatment of sexual dysfunction in HIV+ patients. TAD is metabolised mainly by the CYP3A4 isoform [99], on which TPV acts as an enzyme inducer and RTV as an inhibitor. Due to the conflicting actions expressed by these antiretrovirals, it was necessary to evaluate the effects on various PK parameters of TAD [76]. The authors showed that administration of a single oral dose of TAD 10 mg with the first dose of oral TPV/r 500/200 mg twice daily led to an increase in the AUCinf of TAD by 133%. This is presumably due to the increased in RTV exposure during the first few days of treatment with TPV/r, when the RTV-dependent inhibitory effect on CYP3A4 is likely to occur, and then disappear once TPV/r has reached a steady-state. For these reasons, when it is necessary to take TAD during the first few days of treatment with TPV/r, it is advisable to reduce the dose of TPV/r by 50% until steady-state is reached [76].

The effect of enzyme induction by lopinavir/ritonavir (LPV/r) was also analysed in the study by Hogeland et al. in relation to its potential ability to induce CYP2B6 and UGT1A1.

In particular, the concomitant use of oral LPV/r 400/100 mg twice daily with oral bupropion (BUP) 100 mg daily, an antidepressant used in the treatment of major depression, obesity, asthenia, and smoking cessation, resulted in a significant decrease in Cmax by 57% and AUCinf by 57%, requiring an increase in BUP dosage by up to 100% [82].

The same effects were studied by Jacobs et al. with regard to CYP1A2 as well as UGT isoforms, with the use of oral fosamprenavir (FPV), pro-drug of amprenavir (APV), in combination with oral RTV 700/100 mg twice daily. Co-administration of these antivirals with oral olanzapine (OLZ) 15 mg an atypical antipsychotic, reduced plasma half-life (t_1/2_) of OLZ by an average of 32%. Increasing the dose of OLZ from 10 mg to 15 mg (50%) the AUC returned to the bioequivalence range [30]. The same antiviral combination was co-administered with posaconazole (POS), an antimycotic, in the study by Brüggemann et al.

The authors suggest that the use of FPV alone with the antimycotic agent resulted in a marked decrease of AUC and Cmax of APV, compared to the use of oral FPV boosted with RTV (2.9- and 1.6-fold lower) 700/100 mg twice daily, and therefore unboosted FPV 700 mg twice daily should not be used in combination with oral POS 400 mg once or twice daily [22].

Fichtenbaum et al. [25] investigated potential DDIs between PIs and statins 40 mg. The study population was subdivided into 4 arms; arms 1, 2, and 3 received oral pravastatin (PRAV), simvastatin, and atorvastatin (ATO), respectively, with oral RTV 400 mg + saquinavir (SQV) 400 mg twice daily, while arm 4 received oral pravastatin 40 mg with oral NFV 1250 mg twice daily. Because of RTV-dependent inhibition on CYP3A4, statins that are mainly metabolized by this enzyme (e.g., simvastatin and lovastatin) should be not co-administered with RTV-based HAART, while treatment with ATO should be initiated at doses of 10 mg daily. In contrast, PRAV/NFV did not affect NFV/M8 concentration [25].

In addition, Yu et al. found that exposure to oral pitavastatin (PTV) 4 mg daily decreased by 26% for the concomitant use of oral darunavir (DRV) co-formulated with RTV (DRV/r) 400/100 mg daily, which can alter the activity of transporters involved in PTV uptake and efflux [87].

## 3. Studies Investigating DDIs Between HAART and Co-Administered Drugs in HIV+ Patients

Table 2 shows the main characteristics and results of 14 studies involving HAART and co-administered drugs in HIV+ patients. With regard to the study design, 4 of them are RCTs [69,100,101,102], 6 are referred to as PK studies [103,104,105,106,107,108], 1 is an observational study [109], 1 is referred to as pharmacogenetic (Pgx) study [110] and 2 as DDIs studies [111,112]. These investigations, which altogether enrolled 703 subjects, had in common the main objective of identifying potential DDIs involving one or more drugs used in the HAART regimen that may cause ADEs or changes in the PK profiles of antivirals and co-administered drugs [69,100,101,102,103,104,105,106,107,108,109,110]. In particular, 6 out of 14 [69,100,101,102,107,109] concerned potential DDIs between different drugs belonging to HAART, while 8 out of 14 explored DDIs between drugs contained in HAART and others belonging to different therapeutic classes. Of the latter, 5 studies [103,104,105,108,110] concerned the use of contraceptives, 1 concern the anticancer drug sunitinib [106], 1 the direct oral anticoagulant dabigatran [111], and 1 the statins fluvastatin (FLUV) and PRAV [112].

PIs and NNRTIs have often been studied for their potential involvement in DDIs with other drugs used to treat comorbidities commonly present in PLWH, or prophylactic agents. With regard to PIs, the interaction between LPV/r and contraceptives is well-known. In fact, the Summary of Product Characteristics (SmPCs) of LPV and RTV recommend the use of additional contraceptive methods during therapy with these antivirals due to the risk of reducing the contraceptive effect.

Luque et al. [105] investigated the clinical outcomes potentially associated with the co-administration of oral LPV/r 400/100 mg and intramuscular (IM) depot medroxyprogesterone acetate (DMPA) 150 mg in HIV+ women. The authors found a significant increase in DMPA exposure in patients co-administered with LPV/r. First, PK parameters, such as AUC and Cmax of LPV and RTV were measured when co-administered separately with DMPA. In this case, no significant impact was found. Next, when LPV and RTV were coformulated as LPV/r, the authors found a 46% increase in the AUC and a 66% increase in Cmax of medroxyprogesterone acetate (MPA), the active metabolite of DMPA. These effects were probably due to the inhibitory action of LPV/r on CYP3A4, which is involved in DMPA metabolism, resulting in the risk of menstrual irregularities and abnormal vaginal bleeding [105].

Vogler et al. [103] reported a significant reduction (45%) in plasma levels of transdermal ethylestradiol (EE) 33.9 μg/24 h and an increase (83%) in norelgestromine (NGMN) 203 μg/24 h in patients treated with the oral lopinavir/ritonavir (LPV/r) regimen 400/100 mg twice daily. The authors suggested that increased plasma levels of NGMN could increase the risk of venous thromboembolism (VTE). The hypothesised mechanism is the potential action of LPV and RTV as CYP450 enzyme inhibitors [103].

Another DDI involving PIs and contraceptives is described by DuBois et al. [104]. This study showed that exposure to contraceptives containing oral norethindrone (NETA) 0.35 mg daily, may increase when oral ATV is co-administered with RTV 300/100 mg. In fact, the authors found 50% increase in the AUC and a 67% increase in the Cmax of NETA. Moreover, oral clearance (Cl/F) and volume of distribution (Vd) decreased, suggesting that interaction with RTV increases the bioavailability of NETA, without affecting its half-life in plasma.

Another DDI related to PIs concerns sunitinib. This is reported in the SmPC of RTV, which warns that concomitant use of RTV may increase serum concentrations of sunitinib due to RTV-dependent CYP3A4 inhibition. Rudek et al. [106] investigated ADEs that could result from such DDIs in HIV+ patients with solid or hematological malignancies. In particular, it was found that patients treated with oral sunitinib 50 mg daily in the absence of RTV showed toxicity (grade 3 neutropenia and grade 1/2 diarrhea, mucositis, and fatigue) comparable to that of patients receiving sunitinib 37.5 mg daily concomitantly with oral RTV 25 mg daily. Based on these results, the authors suggested reducing the sunitinib dosage to 37.5 mg in patients receiving RTV [106].

With regard to the NNRTI class, both EFV, and nevirapine (NVP) were examined in combination with contraceptives. These interactions may influence plasma levels of contraceptives, impairing their efficacy and increasing the risk of ADEs related to both the HAART and the contraceptives themselves.

The study conducted by Vieira et al. [108] also evaluated the DDIs between EFV and ENG. The oral EFV-based HAART regimen, containing 600 mg of the NNRTI, reduced the bioavailability of subdermal implant ENG 68 mg, by decreasing its AUC, Cmax and Cmin by 63.4, 53.7 and 70%, respectively. This is because EFV acts as an inducer of the CYP3A4 enzyme. Cases of unwanted pregnancies have been reported in women using ENG while taking an EFV-based HAART, emphasizing the need for caution when co-administering these two drugs [108].

A study by Neary et al. [110] explored potential DDIs between oral EFV 600 mg daily or oral NVP 200 mg twice daily and the subcutaneous implant etonogestrel (ETON) 68 mg. The results indicated that genetic factors, particularly specific polymorphisms in the *CYP2B6* gene, may significantly influence plasma levels of ETON. In particular, the *CYP2B6* 516 G>T genetic variant was identified as a factor capable of influencing EFV concentration, leading to increased plasma exposure. Since EFV is an inducer of CYP3A4, which is responsible for ETON metabolism, a reduction in contraceptive levels may occur following the co-administration of the two drugs. In this study, a 33% reduction in the AUC of ENG was observed in participants homozygous for the T allele compared to those homozygous for the G allele of the *CYP2B6* gene (516 G>T). Similarly, the AUC of levonorgestrel (LNG) was 64% lower in subjects with homozygous TT allele than in those homozygous GG for the same polymorphism. Furthermore, among participants homozygous CC and heterozygous CT for the CYP2B6 polymorphism (983 T>C), there was a 20% decrease in the AUC of ETON, while for LNG there was a 23% decrease in this same group. This evidence suggests a greater decrease in PK exposure to ETON in the presence of CYP2B6 genetic variants, which correlate with reduced metabolism of EFV. This effect could be explained by high concentrations of EFV inducing increased activity and expression of CYP3A4, an enzyme known to facilitate ETON elimination. These DDIs could significantly decrease the efficacy of hormonal contraceptive implants in HIV+ women, thus limiting their use [110].

Several studies have analyzed DDIs between drugs belonging to the HAART regimen. For instance, the research by Fletcher et al. [100] showed that the combination of the oral PI SQV administered 800 mg three times daily, with the oral NNRTI delavirdine (DLV) 600 mg twice daily, and the oral NRTI adefovir dipivoxil (ADV) 120 mg once daily, resulted in a reduction of plasma SQV concentrations by approximately 50% compared to subjects treated with DLV alone. This result indicates a significant interaction between SQV and DLV. Furthermore, the reduction in SQV concentrations occurred exclusively in the groups receiving the combination of DLV and ADV, whereas similar effects were not observed with other PIs, such as oral RTV 400 mg daily, and oral NFV 750 mg three times daily. No synergistic or additive effects were found between LDV and ADV, despite their different mechanisms of action, with disappointing virological results. These ADRs could be attributable to CYP450-mediated mechanisms of metabolism and the activity of transporter proteins such as P-glycoprotein (P-gp) [100].

The study conducted by Sekar et al. [102] found that the addition of oral DRV/r 300/100 mg (oral solution, treatment A) or 400/100 mg (tablets, treatment B2), to a therapeutic regimen including oral NVP 200 mg, resulted in an increase in plasma levels of NVP, as RTV inhibits the enzyme CYP3A4, responsible for its metabolism, as measured through blood samples and therapeutic drug monitoring (TDM). TDM, by recording plasma levels at predetermined intervals, allowed Cmin, Cmax, and AUC to be calculated. However, this increase did not appear clinically significant, and overall, the combination of DRV/r and NVP was generally well tolerated by HIV+ patients, although the study evaluated lower doses than typically recommended, no dose adjustment of NVP is expected even at higher doses [102].

**Figure 1:**
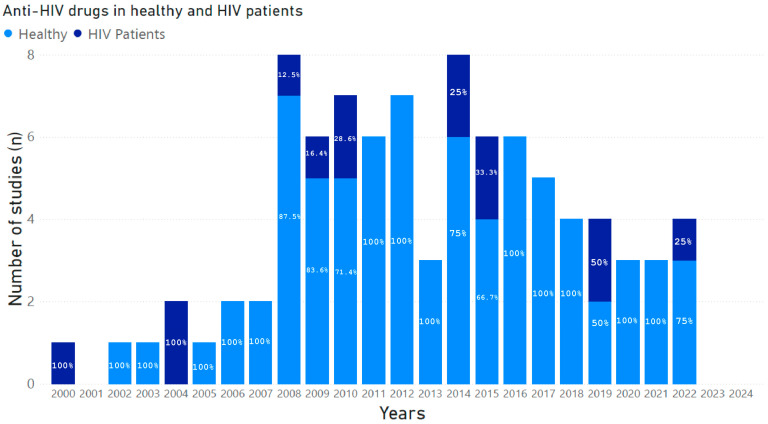
Number of studies published between 2000 and 2024, conducted in healthy volunteers and *HIV*+ patients that investigated potential DDI-related adverse outcomes involving drugs belonging to HAART and drugs co-administered for the treatment of comorbidities. The percentages of studies, calculated from the total number of studies published year by year, are marked within each histogram.

Figure 1 shows the number of studies published during the period 2000–2024, conducted in healthy volunteers and HIV+ patients that investigated potential DDI-related adverse outcomes involving drugs belonging to HAART and drugs co-administered for the treatment of comorbidities.

## 4. Studies Investigating DDIs Between HAART and Co-Administered Drugs in HIV Patients with Coinfection

PLWH are at increased risk of coinfection. Table 3 shows the main characteristics and results of studies that have enrolled HIV+ patients with TB, malaria, or HBV.

Studies reporting data on HIV/HCV coinfection, which are more numerous and mainly involve direct-acting antivirals (DAAs), are described in the next paragraph.

Nineteen studies, involving a total of 2712 patients, among them, 7 were RCTs [113,114,115,116,117,118,119], 8 were referred to as PK studies [120,121,122,123,124,125,126,127], 2 to as DDIs studies [128,129], 1 was a prospective cohort study [130] and 1 was retrospective cohort study [131]. This studies analyzed potential DDIs [127,128] or DDI-related adverse outcomes [118,119,120,129]. Fourteen studies [113,114,115,116,117,119,121,122,123,126,127,128,130,131] have focused on TB, 4 [118,120,124,125] on malaria, and only 1 study on HBV [129].

A common objective of these studies was to investigate potential DDIs between drugs used in PLWH with coinfection. Among the 14 studies in HIV/TB patients, 4 focused on the co-administration of the anti-TB RIF and the anti-HIV EFV demonstrating the efficacy and good tolerability of this drug combination. Lopez-Cortes et al. [115] have observed that it is necessary to increase the oral dose of EFV from 600 mg to 800 mg daily when combined with RIF 120 mg daily because this anti-TB is a strong inducer of the CYP3A4 enzyme. Scarsi et al. [119] have analysed PK of EFV 600 mg daily and RIF 10 mg/kg daily when they are combined with oral LNG 1.5 mg or 3 mg daily, a drug used for long-term contraception. This study showed that both EFV and RIF accelerate LNG metabolism through induction of the CYP3A4 isoform, leading to a reduction in half-life and a decrease in overall LNG exposure. However, this effect did not affect contraceptive efficacy of LNG [119].

Moreover, RIF is known to induce the CYP2B6-mediated EFV 8-hydroxylation pathway, potentially causing an increase in the metabolism of drugs metabolized by this enzymatic isoform, such as EFV [132]. Actually, Ren et al. [123] demonstrated a clear trend towards a reduction in oral EFV 50 mg half-life in children when RIF 10 mg/kg daily was administered together with EFV without changing the Cmin of EFV. This effect on EFV half-life was not evidenced in HIV/TB co-infected even when doubling the dose of RIF [123].

Therefore EFV/RIF co-administration was considered safe both in children and adults HIV/TB co-infected patients [113,123]. Two studies have provided details on safety profile of RIF co-administered with LPV/r, showing that CYP3A4-inducing effect of RIF is reverted by the CYP3A4-inhibiting effect of RTV in the LPV/r formulation [116,122].

Two studies have provided details on the safety profile of RIF co-administered with LPV/r, showing that the CYP3A4-inducing effect of RIF is negated by the CYP3A4-inhibiting effect of RTV in the LPV/r formulation [116,122]. In the study conducted by Zhang et al. [116] the AUC of oral LPV/r (as oral solution) 230/57.5 mg/m^2^ twice daily alone and in combination with RIF 10 mg/kg daily was analyzed in pediatric and adult patients. The AUC of LPV/r decreased more in children than in adults, due to the longer absorption and transit times of LPV and RTV in children. Another bactericidal antibiotic, rifabutin (RFB), has also been shown to interact with the HAART regimen. A study by Narita et al. demonstrated that the combination of oral RFB 300 mg and PIs, including indinavir (IDV) administered 800 mg, 1000 mg, or 1200 mg every 8 h, or 1200 mg twice daily, or NFV (1000 mg or 1250 mg twice daily), is effective in HIV/TB coinfection. Indeed, RFB retains its bactericidal activity, while PIs continue to suppress *HIV* without increasing side effects [128].

Studies focusing on malaria, all conducted in Africa, reported that the most prescribed drugs for HIV/malaria patients are the antimalarial agents artemether-lumefantrine (AL), dihydroartemisinin-piperaquine (DPQ), artesunate/amodiaquine (AS/AQ) and the anti-HIV drugs NVP, EFV, and LPV/r. In particular, the study conducted by Byakika-Kibwika et al. [120] analyzed potential DDIs between oral AL 80/480 mg and oral EFV 600 mg daily or NVP 200 mg daily for 2 weeks, than 200 mg twice daily. The results showed that when AL is taken together with EFV or NVP, patient exposure to the anti-malarian drug is reduced. This could be due to the ability of EFV and NVP to increase CYP3A4 activity, resulting in an accelerated metabolism of drugs metabolized by this enzyme isoform, such as AL [133,134,135]. The authors concluded that this effect could lead to the failure of anti-malarian treatment [120]. This evidence is supported in the study by Scarsi et al. [124] in which the potential DDIs between drugs in the HAART regimen, such as zidovudine (AZT), lamivudine (3TC), and NVP coformulated in a single 200/300/150 mg tablet and oral AS/AQ 80/480 mg, was examined.

In particular, the relationship between the AUC of the metabolite desethylamodiaquine (DEAQ) and the progenitor drug (amodiaquine) was measured. The results revealed that patients taking this HAART regimen had reduced exposure to AS or its metabolite DEAQ due to the ability of NVP to induce CYP3A4 and CYP2B6 enzyme isoforms. Indeed, although to a lesser extent than CYP2C8, these enzymes are involved in the metabolism of the antimalarial agent.

Two studies by Wallender et al. Sevene et al. [118,125], which enrolled 83 and 221 patients respectively, discussed potential DDIs between DPQ 40 mg for the first and 320 mg for the latter study and oral EFV 50 mg, due to EFV-mediated CYP3A4 induction. This led to an increase in piperaquine (PQ) clearance, compromising the efficacy of chemoprophylaxis with dihydroartemisinin-PQ (DHA-PQ) and making the monthly regimen insufficient to prevent malaria. It is suggested that daily administration of low-dose DHA-PQ (320 or 160 mg PQ) might be a promising strategy to overcome the effects of these DDIs. In addition, switching to a DTG-based antiretroviral regimen could improve the clinical outcomes of HIV+ pregnant women [118]. Moh et al. found that the combination of oral AZT 300 mg and oral cotrimoxazole (TMP-SMX) 160/800 mg, caused significant adverse effects, including increased hematological toxicity, such as neutropenia and anaemia. These effects were more common when the drugs were used together rather than separately. This is because AZT inhibits *beta-globin* gene expression, resulting in bone marrow toxicity and a dose-dependent reduction of the neutrophil count. Similarly, TMP-SMX can cause neutropenia and anaemia by inhibiting dihydrofolate reductase in a dose-dependent manner [129].

## 5. Studies Investigating DDIs Between HAART and Co-Administered Drugs Used for the Treatment of HCV in Healthy and HIV/HCV Co-Infected Patients

Chronic HCV infection is a major cause of morbidity and death in PLWH [136]. Indeed, HIV/HCV co-infected patients have a higher risk of developing advanced liver fibrosis and cirrhosis than HCV monoinfected patients. This makes the treatment of HCV infection a priority in PLWH [137]. However, combining antiviral regimens in the co-infected population can be complex, as the therapies may share overlapping PK, especially with regard to metabolism or elimination that may result in DDIs [138].

Twenty-five studies involving 2635 subjects treated with HAART and anti-HCV drugs were analyzed. Seventeen studies enrolled healthy volunteers and 8 patients with HIV/HCV co-infection (Table 4). Of these 25 studies, 10 are RCTs [139,140,141,142,143,144,145,146,147,148], 10 are PK studies [149,150,151,152,153,154,155,156,157,158], 2 are defined as PK-DDI studies [159,160] and one is a retrospective/prospective study [161]. For the remaining 2 [162,163], the study design is not reported (Table 4). Numerous studies have analyzed the PK, safety and tolerability of different antiviral drug combinations. However, they were mainly conducted in healthy volunteers, thus making comparison with studies that evaluated the same drug combination on HIV/HCV co-infected patients very difficult.

As early as 2003 and 2004, Carrat et al. and Poizot-Martin et al. [139,161], evaluated the efficacy and tolerance of anti-HCV treatment with interferon (IFN) or Peg-Interferon (PegIFN) and ribavirin (RBV) in patients with HIV/HCV coinfection. The study by Carrat et al. reported treatment with oral RBV 400 mg twice daily plus subcutaneous PegIFN alpha-2b 1.5 µg/kg once a week or standard subcutaneous IFN alpha-2b 3 million units three times a week for 48 weeks [139]. Poizot-Martin et al. reported the cases of patients who received oral RBV 800 mg, two tablets daily, and Peg-IFN 180 mg once a week or IFN-alpha-2b 3 MU three times a week for at least 6 months and up to 12 months [161].

Both studies reported a reduction in Sustained Virological Response (SVR) in patients taking HAART drugs, in particular PIs. Furthermore, an increase in serious ADEs was observed in HIV+ patients compared to HIV-negative patients, especially when they were taking older NNRTis such as didanosine [139]. In contrast, studies by Chung et al. and Torriani et al. [140,141], evaluating the same treatment regimen with almost identical dosage in co-infected patients, showed that the use or non-use of antiretrovirals and the use or non-use of PIs were not predictive of SVR. However, Chung et al. [140] confirmed the concern about ADEs occurring in regimens including didanosine, probably due to increased intracellular concentrations of its active metabolites.

Notably, over the past two decades, the only standard treatment for HCV-infected patients was PegIFN/RBV, but only a limited percentage of patients managed to achieve SVR. With the subsequent introduction of DAAs into clinical practice (in 2011), the cure rate of chronic HCV increased significantly [164]. DAAs are divided into three main classes based on their targets in HCV: PIs of the non-structural protein 3/4A (NS3/4A), which can inhibit HCV polyprotein processing; NS5A inhibitors, which inhibit viral replication and assembly; and NS5B polymerase inhibitors, which can block *HCV* RNA replication [165]. Various detrimental PK DDIs were identified when DAAs were administered with PIs/r in healthy volunteers but the same DDIs were not investigated in patients.

Hulskotte et al. conducted a randomized, open-label study to evaluate PK interactions between oral boceprevir (BOC) 800 mg, an *HCV* serine protease NS3 inhibitor, taken 3 times daily and PIs/r (ATV/r 300 mg daily, DRV/r 600 mg twice daily, LPV/r 400 mg twice daily each with RTV 100 mg) in 39 healthy adults, demonstrating that such co-administration can result in reduced exposure of both PIs and BOC. However, the treatments were overall well tolerated [142]. The following year, a similar PK study was conducted in 14 HIV/HCV patients taking oral ATV/r 300/100 mg daily and a triple therapy for chronic hepatitis C based on oral telaprevir (TVR) 1125 mg twice daily, a HCV NS3/4A protease inhibitor, PegIFN alpha, and RBV. PK profiles were assessed before and after switching from ATV/r to unboosted ATV (200 mg twice daily). Similarly to what was reported on BOC, the co-administration of TVR with ATV/r resulted in reduced exposure of both drugs compared with administration without the booster [149]. The observed DDIs can be surely explained by the involvement of CYP3A4/5 and P-gp-dependent mechanism of which PIs, BOC, and TVR are substrates and inhibitors. As matter of the fact, the concomitant use of these drugs with several HAART components is not recommended [166]. Since these bidirectional DDI, it is very difficult to assess the relative contribution of each drug to the observed results. Unlike the previous two studies [142,149], Khatri et al. study [151], which consisted of five PK studies of Phase 1 involving 144 healthy volunteers, has shown that the 3D regimen consisting of ombitasvir (25 mg once daily), paritaprevir/ritonavir (150/100 mg once daily), and dasabuvir (250 or 400 mg twice daily)—OBV/PTV/r + DSV)—is not recommended with the evening intake of oral ATV/r 300/100 mg and oral LPV/r 800/200 mg due to higher exposure to PTV or RTV. This may be partially due to the increased daily dose of RTV (200 mg/day for the evening dosing regimen compared with 100 mg/day for the morning dosing regimen) and the resulting inhibition of the metabolism of PTV. In addition, being a substrate of CYP3A, OATP1B1 and OATP1B3, PTV may be subjected to the inhibitory effects on these proteins exerted by ATV and RTV. An increase in total bilirubin levels was identified in the ATV arm, but no serious ADEs were reported.

Similarly, DDI studies were conducted in healthy volunteers also to evaluate the effect of RTV on the PK of an oral dose of grazoprevir (GZR) 200 mg, an NS3/4A protease inhibitor, and oral elbasvir (EBR) 50 mg daily, an NS5A inhibitor, when co-administered with oral ATV/r 300/100 mg daily, oral LPV/r 400/100 mg twice daily, or oral DRV/r 600/100 mg twice daily. These studies, having demonstrated increased exposure to GZR when GZR-EBR are co-administered with PIs/r, suggested that the use of HIV treatment regimens lacking PIs/r should be considered in HIV/HCV-co-infected individuals who need to be treated with these two DAAs [152]. Again, Kosloski et al. [159], which evaluated the PK of oral glecaprevir (GLE) 300 mg, an NS3/4A protease inhibitor, and pibrentasvir (PIB) 120 mg, an NS5A inhibitor, when administered orally alone or in combination with anti-HIV agents, the AUC of GLE was found to be increased (by more than 4-fold) when taken with PIs/r (ATV/r 300/100 mg daily, DRV/r 800/100 mg daily, 400/100 mg twice daily), while PIB concentrations were not affected. In contrast, GLE and PIB exposure was significantly reduced following concomitant intake of oral EFV 600 mg daily. Increases in alanine transaminase occurred in combination with ATV/r [159]. In conclusion, based on the available literature, NS5A inhibitors represent the class of DAAs less affected by the concomitant PI/r intake.

Among NS5A inhibitors, daclatasvir (DCV) has limited influence on CYP3A4 compared with NS3 PIs, making it unable to influence patient exposure to HAART components. In fact, DCV, although a substrate and inhibitor of P-gp, is not a strong CYP3A4 inhibitor [167]. Nonetheless, because DCV is a substrate of CYP3A4, it can be involved in DDIs with potent inhibitors or inducers of the CYP450 system resulting in requirement of dose adjustments [153]. In contrast, co-administration of EFV appears to influence plasma levels of the NS5A and NS3/4A inhibitors. Similar to the study by Kosloski et al. [159], which found a significant reduction in exposure of GLE and PIB when co-administered with EFV, Sabo et al. [154] found a significant reduction in plasma levels of oral faldaprevir (FDV) 240 mg daily, a NS3/4A inhibitor, when co-administered with oral EFV 600 mg daily, consistent with EFV-dependent induction of CYP3A. This decrease could be managed by using the higher dose of the 2 doses of FDV tested in the phase 3 studies [154]. Mogalian et al. [143] found an approximately 50% reduction in exposure of oral velpatasvir (VEL) 100 mg, an NS5A inhibitor, when combined with oral sofosbuvir (SOF) 400 mg, an HCV polymerase inhibitor, and oral EFV 600 mg daily. Of note, in contrast to the aforementioned data, Sabo et al. [154] failed to find clinically significant DDIs when oral FDV was taken with oral DRV/r 800/100 mg daily.

INIs have also been shown to give potential DDIs with antiviral drugs used for HCV treatment. In 2011, Ashby et al. observed no statistically significant differences in the PK parameters of oral RAL 400 mg twice daily when taken alone or with a single oral dose of RBV 800 mg in healthy volunteers [155]. Since RAL is eliminated after UGT1A1-mediated glucuronidation and is a substrate of P-gp [168,169], Joseph et al. [156] evaluated the co-administration of oral FDV 240 mg twice daily, a UGT1A1 inhibitor, and of oral RAL 400 mg twice daily in 24 healthy subjects. Compared to RAL alone, co-administration of FDV resulted in a 2.7-fold and 2.5-fold increase in the mean AUC and Cmax of RAL, respectively. Of note, the incidence of ADEs, especially gastrointestinal disorders, was also higher during combination treatment compared to treatment with RAL alone. RAL is also inhibited by OBV, PTV and DSV [170]. The study by Khatri et al. [157], investigated the effects of combining oral RAL 400 mg twice daily with these inhibitors (PTV/r 150/100 mg daily, OBV25 mg daily, and DSV400 mg twice-daily) in healthy volunteers, showing a 100 to 134% increase in AUC and Cmax of RAL. However, we found only one prospective study that evaluated the PK profile of RAL when co-administered with OBV/PTV/r + DSV regimen in HCV-co-infected PLWH [150]. In particular, this study assessed the PK of oral RAL 400 mg twice daily before and during combined administration of oral OBV/PTV/r 25/150/100 mg once daily + DSV 250 mg twice daily regimen in patients with HIV/HCV co-infection. Contrary to expectations, a decrease in RAL exposure was observed during co-administration of HCV therapy. The authors suggested that this could be due to the rapid elimination of *HCV* induced by the use of potent DAAs and the consequent reduction of inflammation, restoration of hepatocyte function, and drug-metabolising enzyme activities, including UGT1A1 [171]. Furthermore, viral infections, inflammatory responses, and subsequent tissue damage are also known to alter *P-gp* gene expression [172,173]. This emphasizes that the results obtained in healthy subjects can be very different from those found in patients due to disease-specific factors (e.g., comorbidity and therapeutic adherence) that strongly contribute to PK variability.

**Figure 2:**
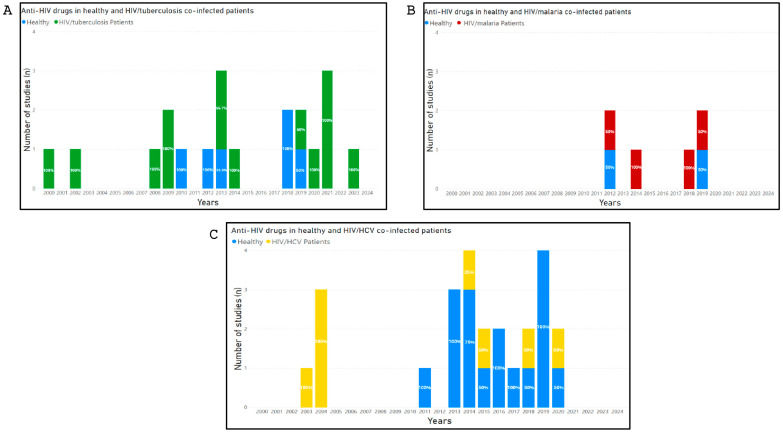
Number of studies published between 2000 and 2024, that investigated potential DDI-related adverse outcomes involving drugs belonging to HAART and drugs co-administered for the treatment of co-infections (malaria, tuberculosis and HCV). The percentages of studies, calculated from the total number of studies published year by year, are marked within each histogram. (Panel **A**) shows the number of studies, conducted in HIV mono-infected and HIV/Malaria co-infected patients, that investigated potential DDI-related adverse outcomes involving drugs belonging to HAART and drugs co-administered for the treatment of malaria. (Panel **B**) shows the number of studies, conducted in HIV/Tuberculosis co-infected patients, that investigated potential DDI-related adverse outcomes involving drugs belonging to HAART and drugs co-administered for the treatment of Tuberculosis. (Panel **C**) shows the number of studies, in healthy volunteers and HIV/HCV co-infected patients, that investigated potential DDI-related adverse outcomes involving drugs belonging to HAART and drugs co-administered for the treatment of HCV.

Studies that investigated potential DDI-related adverse outcomes involving drugs belonging to HAART and drugs co-administered for the treatment of co-infections (malaria, tuberculosis and HCV) were shown in Figure 2.

## 6. Discussion and Conclusions

The introduction of HAART has led to a significant increase in the life expectancy of PLWH. Although this represents an undisputed success of medical-scientific progress, physicians are faced with the complexity of managing patients suffering from several comorbidities and thus undergoing multiple treatments. One of the most relevant consequences is the occurrence of DDI-related ADEs, which were not easily detected during RCTs involving drugs (although commonly used) in these patients. Therefore, it is important to identify and thoroughly investigate clinically relevant DDIs and to define the most appropriate therapeutic approaches.

This is particularly important in HIV co-infected patients. For instance, individuals with HIV/HCV co-infection treated concomitantly with antivirals for HCV and HIV account for about 6% of HIV mono-infected patients worldwide (almost 2 million individuals) [174,175]. Other concerns are HIV+ patients co-infected with malaria or TB that have been enrolled in studies performed in countries where these HIV coinfections are prevalent, such as Africa [113,114,116,118,119,120,121,122,123,124,125,127,129,131] and South-East Asia [119,128,130].

In the present study we reviewed 134 studies published over the last two decades, that collectively emphasize the importance of investigating DDI-related adverse outcomes involving HAART components and drugs co-administered for the treatment of comorbidities in PLWH.

With regard to DDIs involving HAART, most of the studies (84%) were conducted in healthy volunteers, 40% of them reported DDI-related ADEs, including the need to change the drug dosage or the recommendation to avoid (or to pay special attention to) certain combination therapies.

In the early 2000s, PIs and NNRTIs were the main used pharmacological agents. In 2002, Fichtenbaum et al. assessed potential clinically relevant DDIs between statins (oral simvastatin, ATO or PRAV) 40 mg, and the oral PIs SQV 400 mg, RTV 400 mg and NFV 1250 mg in 56 healthy volunteers. The authors concluded that concentrations of simvastatin or ATO significantly increased when combined with SQV/r. Moreover, because of decreased concentration, the authors suggested that it may be necessary to adjust the dosage of PRAV [25].

A potential DDI involving another PI (i.e., IDV) was subsequently studied in 25 HIV+ patients, 12 treated with oral FLUV 20 mg or 40 mg daily, and 13 with oral PRAV 10 mg or 20 mg daily. However, the authors, without measuring plasma concentration of statins, found no changes in lipid parameters [112]. Based on these results it is not possible to establish the real effect of DDIs between PIs and statins.

Potential DDI-related ADEs have been studied between HAART components such as maraviroc (MVC), SQV/r and the antifungal ketoconazole (KCZ), which is a strong inhibitor of the CYPP450 system [17,34]. Regarding MVC, an increased in its plasma levels was reported without any recommendation [17]. No dose adjustment was suggested when SQV/r (1000/100 mg twice daily) was taken with 200 mg KCZ once daily, while using high doses of KCZ (>200 mg/day) were suggested to be avoided [34]. Again, it is not possible to establish the impact of these drug combinations in clinical practice due to the absence of studies enrolling HIV+ patients receiving this therapy.

One of the most important innovations in HIV treatment has been the introduction of INIs, such as RAL and DTG. The increasing complexity of the management of HIV infection necessitated a thorough examination of potential effects and PD of these antivirals in combination with other drugs. In this regard, several studies (including RCTs) have been performed in healthy volunteers, while no research has been conducted in HIV+ patients. It is noteworthy that studies in healthy subjects did not reveal any clinically significant effects of combining RAL with drugs commonly used for the treatment of HIV comorbidities. The study by Iwamoto et al. is an exception, as they report that plasma levels of RAL 400 mg are increased when co-administered with oral OME 20 mg, probably due to an increased bioavailability of oral RAL 400 mg due to an increase in gastric pH [29].

The use of potent inducers of UGT1A1 and CYP3A4, such as CBZ, may cause alteration in the PK of INIs. Song et al. showed a statistically significant decrease in AUC(0-τ), Cmax and Cτ of DTG following CBZ co-administration. This was considered a clinically relevant DDI, so much so that the authors suggested doubling the daily administration of DTG [60]. Other studies have suggested other possible clinically relevant DDIs involving DTG. One of them reported an increase in AUC and Cmax of MET [63] and another a decrease in oral DTG 50 mg absorption, in case of concomitant use of oral calcium 1200 mg and iron supplements 324 mg [56].

Eight reviewed studies explored DDIs between drugs belonging to HAART and drugs belonging to different therapeutic classes in HIV+ patients. Most of them [103,104,105,108,110] concerned the use of contraceptives and demonstrated clinically relevant DDIs. In fact, the SmPCs of LPV and RTV recommend the use of additional contraceptive methods due to the risk of reducing contraceptive efficacy. Similar results were found regarding the combination of EFV with ENG [108] and EFV or NVP with ETON [110]. Notably, this evidence of DDI-related adverse outcome was not marched in studies that enrolled healthy volunteers [18,23,62,65,83].

Apart from studies on the use of contraceptives, the only authors publishing results of ADEs following co-administration of HAART components and other drugs were Rudek et al., who reported grade 3 neutropenia and grade 1/2 diarrhea, mucositis, and fatigue in HIV+ patients with cancer, already treated with RTV, receiving sunitinib due to the RTV-dependent CYP3A4 inhibitory effect [106].

The antivirals used to control anti-HIV and anti-HCV drugs, which are used in multiple therapeutic regimens, are often victims or perpetrators of DDIs involving drug transport proteins or metabolizing enzymes. Data on the treatment of HCV infection in HIV/HCV co-infected patients were scarce until 2004, when data on co-infected populations treated with standard IFN plus RBV or PegIFN plus RBV were reported in three RCTs [139,140,141]. This treatment resulted in lower SVR rates in patients with coinfection compared to those with HCV infection alone [139]. Virological failure was mainly observed in patients taking concomitant HIV-PIs [139,161]. Furthermore, antiviral treatment of both infections often resulted in ADEs that required dose reduction or drug discontinuation, especially when didanosine was also used [139]. Consequently, a warning has been added to the product information for didanosine indicating that RBV is contraindicated in patients also receiving this NRTI [176]. No studies in healthy volunteers were found analyzing the SVR rates and safety profile of the above drug combinations.

Following the introduction in 2011–2012 of first-generation DAAs, TVR and BOC in combination with pegIFN and RBV, SVR rates in patients with HIV and HCV genotype 1 coinfection have improved significantly [177,178]. However, as TVR and BOC are CYP3A4 inhibitors, several evidence on DDIs with drugs included in HAART, (especially the PIs/r), and with other drugs commonly used to treat comorbidities, have accumulated in the following years [142,149]. Thus, given the limited safety data, the Food and Drug Administration (FDA) did not approve the use of TVR and BOC in the co-infected population.

New generation DAAs, which did not require concomitant IFN, revolutionized HCV treatment. They represented an improvement especially for co-infected patients, having eliminated the outcome gap that existed between mono- and co-infections. The combination of SOF, the first approved nucleotide analogue inhibitor of the HCV NS5B polymerase (in 2013), and RBV was the first IFN-free HCV regimen approved for co-infected patients. Furthermore, SOF has a high barrier to resistance, compared to previous DAAs [179], and as it is neither induced nor inhibited by CYP enzymes, PK studies have shown little or no DDIs with a wide range of antiretroviral agents. However, there are some DDIs related to intestinal P-gp, so it should not be co-administered with drugs inducing P-gp, such as RIF [180]. Subsequently, other HCV PIs were introduced, such as paritaprevir, GZR, GLE, as well as other NS5A inhibitors, including ledipasvir, DCV and VEL. As these drugs are always administered in multiple therapeutic regimens several DDI-related clinically relevant outcomes have been shown, especially involving PIs [151,152]. Moreover, according to the available literature, co-administration of PI/r and EFV also seems to influence the plasma levels of the newer NS3/4A and NS5A inhibitors.

Plasma exposure to RAL also appears to be influenced by anti-HCV drugs, depending on whether it is measured in healthy volunteers or in co-infected patients. In this regard, it should be emphasized that the studies that have evaluated DDIs between drugs included in HAART and DAAs, differently by those on the obsolete HAART-IFN+RBV regimen, are almost all conducted in healthy volunteers.

Most of the reviewed studies report inductive or inhibitory effects of drugs included in HAART on enzyme isoforms, especially CYP3A4 and UGT1A1.

For instance, PIs/r, by inhibiting CYP3A4, may increase exposure to drugs commonly used for contraception such as DMPA and NGMN, for erectile dysfunction such as TAD, or for the treatment of co-infections such as PRB/r and GZR. DTG, on the other hand, by inhibiting the organic cationic transporter OCT2, may increase plasma levels of the hypoglycaemic MET.

On the other hand, the drugs mainly involved in enzyme induction are PIs/r such as FPV/r and LPV/r and TPV/r which, by inducing CYP1A2 and UGT1A1 isoforms respectively, can reduce exposure to OLZ, BUP and DTG. In addition, EFV, by inducing CYP3A4, may reduce levels of LNG, ENG, ETON, AL DHA-PQ and GZR.

Notably, drugs belonging to HAART are themselves substrates of the aforementioned enzymes. As a consequence, for example, plasma levels of CAB may be reduced by the inducing effects of RIF on CYP3A4 and UGT1A1, while increased exposure to RAL may be due to inhibitory effect exerted by FDV on UGT1A1.

In conclusion, the data available in the literature suggest that the management of both mono-infected and co-infected PLWH requires that DDI-related outcomes should not be underestimated.

Unfortunately, there is often lack of congruence between studies both in terms of number, drugs involved, and results obtained. This represents a crucial issue from the perspective of clinical pharmacology. In fact, some studies (actually most of those reviewed here), presenting data on the mechanism of action and presumed clinical relevance of potential DDI-related ADEs related to, were conducted in healthy volunteers without being replicated in HIV+ patients.

Infrequently have the same drug combinations been studied in both healthy subjects and patients. However, as factors such as inflammatory status, loss of activity of drug-metabolizing enzymes, and reduced adherence to therapy may influence exposure to HIV and HCV antivirals, the predicted DDIs in HIV/HCV populations may not be the same as those observed in studies with healthy participants. This hampered the evaluation of the impact of DDIs to define the most appropriate treatment in PLWH.

## Figures and Tables

**Table 1:** Main characteristics and results of the studies, published over the last two decades, that have investigated DDIs between HAART and co-administered drugs in healthy subjects. The drugs were orally administered in all studies with the exception of Walimbwa et al. [68] in which AS/AQ were injected.

References	Study Design	Country	Patients (n)	Sex (F%)	Range Age (Years)	Median Age(Years)	Drugs Involved	Follow-Up	Outcomes	Main Results and Conclusion
Abel et al. [17] (2008)	RCT with placebo	EU	80	22.5	18–45	NA	TPV, SQV, KCZ, MVC	7 and 10 days after the final dose of study medication	Influence of CYP3A4 inhibitors on the PK of MVC	CYP3A4 inhibitors can increase MVC exposure. Adjustment of the MVC dose during co-administration with SQV can compensate this effect. TPV/r does not affect PK of MVC.
Anderson et al. [18] (2011)	RCT with placebo	EU	20	100	18–38	NA	RAL, EE, NGM	28 days	Effect of RAL on the PK of the estrogen and progestin components of NGM-EE	RAL has no clinically significant effect on the PK of EE and NGM and the co-administration is generally well tolerated. No dose adjustment are required.
Ankrom et al. [19] (2021)	RCT	USA	14	100	50–64	55.5	ISL (MK-8591), LNG/EE	NA	DDIs between ISL and the COC LNG	Co-administration of ISL (MK-8591) with LNG/EE doesn’t require dose adjustment.
Blonk et al. [20] (2012)	RCT	EU	18	50	18–55	NA	Ginkgo biloba, RAL	36 days	Effect of ginkgo biloba on the PK of RAL	Ginkgo biloba does not decrease RAL exposure. No serious adverse events are recorded.
Blonk et al. [21] (2016)	RCT	EU	24	45.8	18–53	NA	RAL, CIT	NA	DDIs and tolerability of concomitant administration of CIT and RAL	RAL does not impact the PK of CIT or its metabolite, desmethylcitalopram. CIT does not significantly alter the PK of RAL. The combination is well tolerated, allowing for administration without the need of dose adjustment.
Brüggemann et al. [22] (2010)	RCT	EU	24	41.7	18–55	NA	POS, FPV	17 days	Interaction between FPV/RTV and POS	Due to PK reasons, FPV should not be used concomitantly with POS.
Elliot et al. [23] (2018)	RCT	Africa	14	100	19–35	31	EE/LNG with ATV/COBI	NA	PK of EE/LNG co-administered with ATV/COBI	The findings indicated that co-administering EE/LNG with ATV/COBI does not significantly alter LNG concentrations but results in a 25% reduction in EE C24 levels.
Fang et al. [24] (2008)	RCT	USA	20	NA	18–48	NA	NFV, OME	NA	Effect of OME on the multiple-dose PK and safety of NFV	OME/NFV combination can decrease the levels of NFV and its active form.
Fichtenbaum et al. [25] (2002)	RCT	USA	56	59	19–56	32	PIs, statins	Days 1–4 and 15–18	DDI between PIs and statins	Simvastatin should be avoided, and caution should be exercised when using ATO in individuals taking RTV and SQV. It may be necessary to adjust the dosage of pravastatin when it is is used concomitantly with RTV and SQV, while no dose adjustment is required in combination with NFV.
Garrison et al. [26] (2017)	RCT with placebo	EU	42	NA	NA	NA	BIC/FCT/TAF and LDV/SOF or SOF/VEL/VOX	NA	DDIs between EMPA and verapamil, ramipril, or digoxin	No clinically significant DDIs were observed.
Huang et al. [27] (2012)	RCT with placebo	USA	Tot: 51 -G1: 39 -G2: 12	-G1: 74–G2: 92	18–60	NA	RTV-boosted BILR 355, 3TC/ZDV	days 7 and 14	Effect of BILR 355 on 3TC/ZVD	The simultaneous use of BILR 355 with 3TC/ZDV leads to a slight reduction in BILR 355 exposure and a 45% rise in 3TC exposure.
Iwamoto et al. [28] (2008)	RCT with placebo	USA	28	0	18–41	NA	RTV, EFV, RAL	NA	Effects of RTV and EFV on the PK of RAL	No dosage adjustment is necessary when RAL is coadministered with RTV or EFV.
Iwamoto et al. [29] (2009)	RCT with placebo	USA	14	50	22–51		OME, RAL	NA	Effects of OME in population taking RAL	RAL plasma levels increased when it is coadministered with OME in healthy individuals, likely due to enhanced RAL bioavailability resulting from elevated gastric pH.
Jacobs et al. [30] (2014)	RCT	EU	24	54.2	26–50	42	FPV/r, OLZ	NA	Effect of FPV/r on the PK of OLZ	FPV/r 700/100 mg b.i.d. seems to accelerate the metabolism of OLZ. Therefore, is suggest an increase of the OLZ dosage by 50% in case of co-administration with a RTV-boosted PI.
Jann et al. [31] (2012)	RCT	USA	24	37.5	NA	NA	VNF XR/IDV, desvenlafaxine XR/IDV	NA	Effects of VNF XR capsules and desvenlafaxine XR upon IDV PK	VNF XR and desvenlafaxine XR do not affect the metabolism of IDV. There is no evidence of a PK DDIs between VNF XR and IDV, nor between desvenlafaxine XR and IDV.
Jiang et al. [32] (2013)	RCT with placebo	Asia	17	0	NA	NA	CLO, ITR, EFV	8 weeks	Effects of CLO and ITR on the disposition of EFV	CLO pretreatment decreases AUC, C(max) and Ae for 8,14-dihydroxyefavirenz, compared with placebo.
Justesen et al. [33] (2003)	RCT	EU	18	0	23–30	NA	APV, DLV	On day 10 and day 20	Evaluate safety and PK interaction between APV and DLV	APV significantly enhances the metabolism of DLV by inducing hepatic CYP3A4 activity. Conversely, DLV inhibits the metabolism of APV. Therefore, combining APV and DLV twice daily is discouraged when relying on DLV for its ARV effect.
Kaeser et al. [34] (2009)	RCT	EU	42	4.8	19–62	NA	KCZ and RTV-boosted SQV	15 to 21 days after the last dose	DDI study of KCZ and RTV-boosted SQV	No dose adjustment is necessary for SQV/RTV (1000/100 mg twice daily) when taken alongside 200 mg of KCZ once daily. However, using high doses of KCZ (>200 mg/day) is not advised in this combination.
Kakuda et al. [35] (2009)	RCT	USA	40	12.5	-G1: 19–55 -G2: 19–54	42	ETR, TDF	NA	PK interaction between ETR and TDF	No dosage adjustment is required when ETR and TDF are taken together.
Kakuda et al. [36] (2014)	RCT	USA	Tot: 32 -G1 (RFB/ETR): 16 -G2 (ETR/CLR): 16	3.1	18–55	NA	ETR, RFB or CLR	(A) 14–21 days (B) 8–13 days	Interaction between ETR and RFB or CLR	ETR and CLR can be taken together without changes in dosage.
Kasserra et al. [37] (2011)	RCT	USA	150	50.7	18–55	NA	VVC, ATV, DRV, FPV, IDV, NFV, SQV, TPV, RTV, LPV, ZDV/3TC, TDF	29–35 days	Interaction of VVC with other ARV agents (ATV, FPV, IDV, NFV, SQV, DRV, TPV)	No adjustments in dosage or monitoring of VVC levels are required when it is administered alongside any of the ARV drugs
Kearney et al. [38] (2006)	RCT	USA	27	59	19–59	NA	TDF, LPV/r	14 days	PK and safety of TDF on coadministration with LPV/r	The concurrent use of TDF and LPV/r leads to higher steady-state levels of TFV, likely due to enhanced absorption. However, this rise is considered clinically insignificant.
La Porte et al. [39] (2005)	RCT	USA	8	37.5	18–57	33	IDV/r, didanosine	NA	PK interaction between IDV/r and didanosine	The daily dose of 400 mg enteric-coated DDL and 1200/400 mg of IDV/r once daily at breakfast do not reduce the absorption levels of either DDL or IDV. Therefore, this regimen can be taken with food.
Luo et al. [40] (2022)	RCT	Asia	36	25	18–55	NA	HS-10234 (novel prodrug of TFV) and FCT	8 days after the last dose	PK Interactions Between HS-10234 and FCT	No dose adjustments are advised when HS-10234 is combined with FCT.
Mallikaarjun et al. [41] (2016)	RCT with placebo	USA	55	33	18–45	NA	DLM, RTV, RIF, TFV, EFV, LPV/r	NA	DDI between DLM with ARV drugs or antituberculosis	When DLM is taken together with ARVs or antituberculosis medications, it does not exhibit any clinically significant DDIs in healthy individuals.
Matthews et al. [42] (2018)	RCT with placebo	EU	14	NA	19–55	NA	MK-8591 and DOR	NA	PK of MK-8591 and DOR after co-administration	When MK-8591 and DOR are administered together, no clinically significant differences in PK are observed.
Moltó et al. [43] (2021)	RCT with placebo	EU	15	50	23–31	28	COBI, GHB	NA	DDI between GHB and COBI	The study does not identify any PK or PD DDI between COBI and GHB.
Morris et al. [44] (2012)	RCT	USA	34	41.2	18–55	NA	PA, RTV, DHA	NA	DDI between PA and RTV	RTV and PA, when used together, affect the exposure levels of artesunate, DHA, and RTV itself.
Neely et al. [45] (2010)	RCT	USA	19	65	24–51	28,6	RAL, ATV	NA	PK and pharmacogenomics of once-daily RAL and ATV	Although the production of plasma RAL-glucuronide is decreased, the RAL plasma levels showed only minor (statistically insignificant) increases when administered once daily with ATV.
Patel et al. [46] (2011)	RCT	USA	28, 12	0. 14	NA	NA	S/GSK1349572, antacids (maalox advanced maximum strength), OME and multivitamins	NA	PK of S/GSK1349572 with acid-reducing agents and multivitamins	S/GSK1349572 (DTG) can be taken together with proton pump inhibitors and multivitamins without adjusting the dose. However, it should be taken either two hours before or six hours after antacids.
Pene Dumitrescu et al. [47] (2022)	RCT	USA	17	100	18–50	NA	GSK3640254, EE, LNG	14 days	PK DDIs between the HIV-1 maturation inhibitor GSK3640254 and COC	The coadministration of ethinyl estradiol/levonorgestrel with GSK3640254 does not impact the pharmacokinetics or pharmacodynamics of ethinyl estradiol and levonorgestrel in healthy female subjects. No significant tolerability issues are observed.
Ramanathan et al. [48] (2010)	RCT	USA	36	NA	18–45	NA	RTV-boosted EVG (EVG/r) and MVC	NA	PK DDI between RTV-boosted EVG and MVC	When EVG and RTV are co-administered, their PK remain unchanged, while MVC exposures are 2 to 4 times higher, probably due to RTV-mediated inhibition of CYP3A and P-gp. It is recommended to reduce the dose of MVC to 150 mg. Headache is the most reported adverse event related to all treatments.
Ramanathan et al. [49] (2008)	RCT	NA	34	NA	18–45	NA	EVG, ETR	NA	PK of EVG and ETR following coadministration of RTV-boosted EVG (EVG/r) and ETR	No significant DDI between EVG/r and ETR are observed.
Ross et al. [50] (2016)	RCT with placebo	USA	12	25	18–65	NA	DTG, DCV	NA	PK interactions between DTG and DCV	DTG and DCV can be used together without requiring dose adjustment.
Rudd et al. [51] (2021)	RCT	USA	12	58	18–55	39	ISL, DTG, TDF	NA	DDI between ISL, DTG and TDF	No significant changes in the PK of ISL, DTG, or TDF are observed when these drugs are administered together. ISL demonstrates good tolerability whether given alone or in combination with DTG and TDF.
Sanchez et al. [52] (2020)	RCT	USA	14	35.7	19–55	NA	DOR and MET	NA	MET PK after coadministration with DOR	DOR does not significantly alter the PK of MET. MET is well tolerated when administered either alone or with DOR.
Schöller-Gyüre et al. [53] (2008)	RCT with placebo	EU	19	36.8	18–55	49	ETR, RAN, OME	31 days	PK of ETR (TMC125) co-administered with RAN and OME	ETR can be safely used together with proton pump inhibitors and H(2) antagonists without requiring dose adjustments.
Schöller-Gyüre et al. [54] (2008)	RCT	EU	16	0	36–55	42	ETR, MTD	NA	Evaluation of concomitant administration of MTD and TMC125	The concurrent use of ETR and MTD is generally safe and well tolerated ETR does significantly affect the pharmacodynamics of MTD. Therefore, no changes in MTD dosage are required.
Sekar et al. [55] (2010)	RCT	USA	27	NA	33–55	30	DRV/RTV, RFB	NA	PK of DRV/r and RFB	DRV/r may be administered together with RFB, if the dosage of RFB is adjusted to 150 mg, alongside with increased vigilance for possible RFB-related adverse events.
Song et al. [56] (2015)	RCT	USA	24	42	18–65	NA	DTG, mineral supplements	NA	PK of DTG administered with mineral supplements	DTG at a dosage of 50 mg is well tolerated when taken with 1200 mg calcium carbonate or 324 mg ferrous fumarate. Under fasted condition, DTG should be taken two hours before or six hours after calcium or iron supplements.
Song et al. [57] (2014)	RCT	USA	12	0	20–65	36,5	EFV, TPV/r, DTG	NA	Effects of EFV and TPV/r on the PK of DTG	DTG should be administered at an increased dose of 50 mg twice daily when used in combination with EFV or TPV/r.
Song et al. [58] (2011)	RCT	USA	24	12	18–61	NA	ATV, ATV/r, DTG (S/GSK1349572)	NA	Effect of ATV and ATV/r on the PK of S/GSK1349572	The pairing of S/GSK1349572 with ATV/r or ATV is generally well tolerated, with all adverse events being mild or moderate. The most common adverse event is ocular icterus.
Song et al. [59] (2012)	RCT	USA	24	58.3	NA	NA	DTG, food	7 to 14 days	Food effect on the PK of DTG	Consuming food slightly raises DTG exposure from 33% to 66%, depending on the fat content of the meal. This is not expected to affect safety.
Song et al. [60] (2016)	RCT	USA	16	87.5	18–65	NA	CBZ, DTG	7 to 14 days	Effect of carbamazepine on PK of DTG	CBZ greatly lowers the levels of DTG. DTG-naive individuals, who are also taking CBZ, should receive 50 mg DTG twice daily instead of once daily, which is the recommended dose for other strong inducers of UGT1A/CYP3A enzymes.
Song et al. [61] (2013)	RCT with placebo	USA	12	58.3	23–38	NA	Prednisone, DTG	10 days	Effect of prednisone on the PK of DTG	The use of DTG at 50 mg per day and prednisone at 60 mg per day, followed by a 5-day cone, is well tolerated among healthy participants.
Song et al. [62] (2015)	RCT with placebo	USA	16	100	NA	NA	DTG, NGM/EE	7 to 14 days	DTG effect on the PK of NGM/EE	DTG does not impact the PK or PD of NGM/EE. Therefore, no dose adjustment is required.
Song et al. [63] (2016)	RCT	USA	15	27	18–65	NA	DTG, MET	NA	Effect of DTG on PK of MET	DTG has been found to substantially elevate MET plasma levels, partly due to inhibition of OCT2. To ensure optimal glycemic control when initiating or discontinuing DTG in patients taking MET, adjusting the MET dosage is advised.
Song et al. [64] (2010)	RCT	USA	15	6	20–58	**39**	S/GSK1349572, TFV, TDF	NA	DDI between S/GSK1349572, TFV and TDF	S/GSK1349572 and TDF are well tolerated overall, with few adverse events recorded. They can be coadministered without requiring any dose adjustments.
Trezza et al. [65] (2017)	RCT	USA	20	100	18–39	NA	CAB, LNG, EE	NA	PK DDI between CAB and LNG/EE	Repeated doses of oral CAB do not show any notable impact on the PK or PD of LNG and EE.
Van Luin et al. [66] (2009)	RCT	EU	24	0	20–52	NA	RAL, LTG	NA	Effect of RAL on the glucuronidation of LTG	RAL has no effect on the glucuronidation process of LTG.
Vourvahis et al. [67] (2012)	RCT with placebo	USA/EU	32	NA	18–55	NA	LRV, RAL, MVC	NA	PK Effects of coadministration of LRV with RAL or MVR	LRV demonstrated overall good tolerability and seems compatible for concurrent use with RAL or MVC without requiring dose adjustments
Walimbwa et al. [68] (2019)	RCT	Africa/EU	48	NA	≥18	NA	DTG, AL or AS/AQ	NA	DDI between DTG and AL or AS/AQ	DTG does not affect the levels of AL or AS/AQ significantly. Standard doses of these antimalarial regimens should be maintained when DTG is taken.
Weening et al. [69] (2008)	RCT	USA	35	NA	22–68	NA	RAL, TFV	NA	DDI between RAL and TFV	Co-administration of RAL and TDF does not change the PK of either drug, which can therefore be co-administered without dose adjustment.
Zhou et al. [70] (2006)	RCT	USA	32	50	18–65	NA	TBV, 3TC, ADV	NA	PK of TBV with 3TC or ADV	The results support the use of combination therapy or switch between TBV, 3TC, and ADV in the treatment of chronic hepatitis B virus infection.
Zhu et al. [71] (2017)	RCT	USA	32	12	18–45	29	VRC, ATV/r	NA	PK and DDI between VRC and ATV/r	Co-administration of VRC and ATV/r is not recommended unless the potential benefits outweigh the risks.
Zhu et al. [72] (2015)	RCT	USA	36	18	18–49	30	BMS-663068, ATV, RTV, ATV/r	NA	PK DDI between BMS-626529 and ATV/r	Comparing BMS-663068 alone and in combination with ATV/r, in combination the maximum concentration (Cmax) and (AUC) of BMS-626529 in plasma increases. Co-administration with RTV results in a 53% increase in Cmax and 45% increase in AUC of BMS-626529. Systemic exposures of ATV and RTV remained stable whether BMS-663068 is administered with ATV/r. The addition of ATV to the combination of BMS-663068+RTV does not further increase the exposure to BMS-626529.
Zhu et al. [73] (2011)	RCT	USA	56	NA	18–50	NA	ATV/r, OME	NA	Effect of OME on PK of multiple dose ATV/r	A daily dose of 20 mg OME has a significantly milder impact on the PK of ATV compared to the previously noted effects of 40 mg OME.
Blonk et al. [74] (2015)	Crossover CT	EU	24	54.2	18–55	NA	RAL, ATO	14 days	Influence of ATO on the PK of RAL and viceversa	ATO 20 mg does not significantly impact the PK of RAL, and RAL similarly does not affect ATO. Both drugs are well tolerated when taken together and do not require dose adjustments.
Ford et al. [75] (2017)	Crossover CT	EU	15	33	21–65	NA	RIF, CAB	10 to 14 days	Effect of RIF on the Single-Dose PK of oral CAB	Co-administration of CAB with RIF do not affect the maximum plasma concentration of CAB. RIF induced the metabolism of CAB, resulting in an increased clearance and a significant reduction in CAB exposure. RIF is also expected to increase the clearance of CAB when administered as a long-acting injectable drug. Therefore, the concomitant use of RIF with the oral, long-acting formulations of CAB is not currently recommended.
Garraffo et al. [76] (2011)	Crossover CT	NA	17	0	20–40	26	TPV/r, TAD	19 days	Effects of TPV 500 mg and RTV 200 mg combination on the PK of TAD 10 mg	DDIs between a single dose of TAD and TPV/r affects the PK of TPV/r by slightly decreasing patient exposure both during initial dosing and at steady state. Co-administration of TAD significantly increase exposure when given with the initial dose of TPV/r, but this effect diminishes once steady state of TPV/r is achieved. Although ARV activity may remain unaffected, initial TAD doses should be reduced during the initiation of TPV/r therapy, with the option to resume the full dose once steady state is attained.
Kakuda et al. [77] (2014)	Crossover CT with placebo	USA	Tot: 30 -G1: 14 -G2: 16	0	-G1: 21–49 -G2: 18–46	-G1: 34 -G1: 31	ETR, digoxin	14 days	Effect of single- and multiple-dose of ETR on digoxin in healthy subjects	The AUC0–8 h of digoxin showes a slight increase when coadministered with ETR.
Wire et al. [78] (2021)	Crossover CT	USA	14	NA	NA	NA	FTR, MVC	NA	PK DDIs between FTR and MVC	The simultaneous use of FTR and MVC does not lead to significant alterations in the exposure levels of MVC or FTR. These drugs can be given together without requiring any dose adjustments.
Dooley et al. [79] (2012)	Phase 1 PK DDI trial with placebo	EU	37	8	19–62	44	BDQ, EFV	A dose of 400 mg once daily for two weeks followed by 200 mg thrice weekly	DDI between BDQ and EFV	Single-dose BDQ is well tolerated with steady-state EFV.
Dooley et al. [80] (2013)	Phase I PK DDI study	USA	21	2	18–55	NA	DTG, RIF, RFB	NA	Effect of RIF or RFB on the PK of DTG	Regimens including twice-daily DTG and RIF or DTG and RFB once-daily could offer a novel therapeutic option for patients needing simultaneous management of HIV and TB.
Ford et al. [81] (2019)	Phase I, single centre, open label, fixed-sequence, two period study	USA	15	0	NA	NA	RFB, CAB	days 14 and 28	Effects of RFB on the PK of oral CAB	Oral CAB can be safely used alongside RFB without the need of dose adjustment.
Hogeland et al. [82] (2007)	Open-label, sequential design, three phases	USA	12	16.6	22–40	NA	LPV/r, BUP	Every day for 2 weeks	Effect of LPV/r and BUP	The simultaneous use of LPV/r and BUP leads to reduced levels of BUP and its active metabolite hydroxybupropion, potentially requiring a doubling of the BUP dose.
Kearney et al. [83] (2009)	Open-label, fixed-sequence, PK DDI study	USA	20	100	19–45	NA	TDF, HCs (i.e., NGM-EE oral contraceptive)	28 days	Effect of TDF for PK of HCs	No clinically significant DDI between TDF and NGM-EE occurred. Both drugs are well tolerated when coadministered. TDF is unlikely to affect the PK of HCs.
Khalilieh et al. [84] (2017)	Open-label, two-period, two-treatment, fixed-sequence, PK study with placebo	USA	16	50	20–45	NA	DOR, ATO	NA	DOR-ATO DDIs	DOR does not significantly impact the PK of ATO.
van der Lee et al. [85] (2007)	Open-label, multiple-dose, two-arm, two-sequence with placebo	EU	26	69.2	18–65		PAX, FPV/r	NA	PK DDI between PAX and FPV/r	Combining FPV/r with PAX leads to a notable reduction in PAX levels, decreasing exposure by 55%.
Weiner et al. [86] (2014)	Open-label, fixed-sequence, three-period, PK study	USA	21	49.2	25–39	30	RPT, RAL	NA	PK DDIs between RPT and RAL	The combination of RAL and RPT is well tolerated.
Yu et al. [87] (2014)	Single-centre, open-label, multi-dose, fixed-sequence, PK study	USA	28	25	NA	NA	DRV, RTV, DRV/r, PTV	NA	PK interaction of DRV/r and PTV	DRV/r reduces the overall exposure to PTV by 26%, with similar peak exposures observed. PTV does not affect the PK of DRV or RTV. Therefore, DDI between PTV and DRV/r are negligible.
Brooks et al. [88] (2018)	Single-center, open-label, fixed-sequence, DDI study	USA	4	75	21–44	NA	DTG, INH, RPT	22, 26, 30, 33, and 40 days	PK DDIs between INH and RPT with DTG	The concurrent administration of DTG with once-weekly INH-RPT caused severe and unforeseen toxicities due to the release of endogenous cytokines.
Gordon et al. [89] (2016)	Open-label, single-sequence DDI study	USA	16	NA	NA	NA	COBI, DABI	NA	Effect of COBI on DABI	COBI Increases the exposure and the anticoagulant effect of DABI.
Khalilieh et al. [90] (2020)	Open-label, multiple-dose, DDI study	USA	14	50	20–59	35	DOR and MTD	14 days	DDI between DOR and MTD	Co-administration of DOR/MTD has no significant impact on the PK properties of either. Overall, it is well tolerated. No serious adverse effects, and no participant discontinued treatment.
Khalilieh et al. [91] (2018)	Open-label, 2-period, fixed-sequence, DDI study	USA	18	16.7	20–55	NA	RFB, DOR	14 days	Effect of RFB on DOR	DOR can be taken together with RFB if the DOR dosage is increased from 100 mg once a day to 100 mg twice a day.
Majeed et al. [92] (2019)	Open-label, two cohort fixed-sequence, DDI study	USA	36	100	18–45	NA	COBI with ATV or DRV and drospirenone/EE	NA	DDI between COBI-boosted ARV regimens and HCs	Clinical monitoring of drospirenone-related hyperkalemia is recommended with DRV+COBI. ATV+COBI should not be used in combination with drospirenone.

Abbreviations: 3TC, lamivudine; ABC, abacavir; ADV, adefovir dipivoxil; AL, artemether/lumefantrine; APV, amprenavir; ARV, antiretroviral; AS/AQ, artesunate-amodiaquine; ATO, atorvastatine; ATV, atazanavir; ATV/COBI, atazanavir/cobicistat; ATV/r, atazanavir/ritonavir; AUC, area under the concentration; AV, antiviral; BDQ, bedaquiline; BIC, bictegravir; BOC, boceprevir; BUP, bupropion; CAB, cabotegravir; CBZ, carbamazepine; CIT, citalopram; CLO, clopidogrel; CLR, clarithromycin; C(max), maximum concentration; COBI, cobicistat; COC, combination oral contraceptive; DABI, dabigatran; DCV, daclatasvir; DDI, drug-drug interaction; DHA, dihydroartemisinin; DLM, delamanid; DLV, delavirdine; DOR, doravine; DRV, darunavir; DRV/r, darunavir/ritonavir; DSV, dasabuvir; DTG- (S/GSK1349572) dolutegravir; EBR/GZR, elbasvir/grazoprevir; EE, ethinyl estradiol; EFV, efavirenz; EMPA, empaglizofin; ETR, etravirine; EU, Europe; EVG, elvitegravir; FCT, emtricitabine; FDV, faldaprevir; FPV, fosamprenavir; FPV/r, fosamprenavir/ritonavir; FTR, (BMS-663068) fostemsavir; GHB, γ-hydroxybutyric acid; HCs, Hormonal contraceptives; HCV, hepatitis C Virus; HIV, human immunodeficiency virus; IDV, indinavir; IDV/r, indinavir/ritonavir; INH, isoniazide; ISL(MK-8591), islatravir; ITR, itraconazole; KCZ, ketoconazole; LDV/SOF, ledipasvir plus sofosbuvir; LNG, levonorgestrel; LPV, lopinavir; LPV/r, lopinavir/ritonavir; LRV, lersivirine; LTG, lamotrigine; MET, metformin; MTD, methadone; MVC, maraviroc; NA, not available; NFV, nelfinavir; NGM, norgestimate; NVP, nevirapine; OBV-PRV, ombitasvir-paritaprevir; OLZ, olanzapine; OME, omeprazole; PA, pyronaridine/artesunate; PAX, paroxetine; PD, pharmacodynamic; P-gp, p-glycoprotein; PI, protease Inhibitor; PK, pharmacokinetic; POS, posaconazole; PTV, pitavastatin; RAL, raltegravir; RAN, ranitidine; RFB, rifabutin; RIBA, ribavirin; RIF, rifampin; RPT, rifapentine; RPV, rilpivirine; RTV, ritonavir; SOF/VEL/VOX, sofosbuvir/velpatasvir/voxilaprevir; SQV, saquinavir; TAD, tadalafil; TAF, tenofovir/alafenamide; TBV, telbivudine; TDF, tenofovir disoproxil fumarate; TFV, tenofovir; TPV, tipranavir; TPV/r, tipranavir/ritonavir; TVR, telaprevir; USA, United States of America; VNF, venlafaxine; VRC, voriconazole; VVC, vicriviroc; XR, extended-release; ZDV, zidovudine, ZDV/3TC, zidovudine/lamivudine.

**Table 2:** Main characteristics and results of the studies, published between 2000 and 2024, investigating DDIs between HAART and co-administered drugs in HIV+ patients. The drugs were orally administered in all studies with the exception of Vogler et al. [104] in which NGMN and EE were used as a transdermal patch, Luque et al. [106] in which DMPA was intramuscularly administered, Ruxrungtham et al. [108] in which ENF was used by subcutaneous injection. Vieira et al. [109] and Neary et al. [111] used ENG and ETON as a subcutaneous contraceptive implant.

References	Study Type	Country	Patients (n)	Sex (F%)	Range Years	Median (Years)	Drugs Involved	Follow-Up	Outcomes	Main Results and Conclusions
Fletcher et al. [100] (2000)	RCT	USA	Tot: 37 -G1(SQV/r+DLV): 6 -G2(SQV/r+ADV): 7 -G3(SQV/r+ADV + DLV): 5 -G4(SQV/NFV+DLV): 7 -G5(SQV/NFV+ADV): 6 -G6(SQV/NFV DLV+ADV): 6	-G1: 50 -G2: 25 -G3: 50 -G4: 25 -G5: 25 -G6: 25	NA	39.5	SQV/r, SQV/NFV, DLV, ADV,	NA	Evaluation of the steady-state concentrations of SQV, RTV, NFV, DLV and ADV in combination drug regimens	SQV concentrations does not vary when used in combination with RTV or NFV. The addition of ADV to DLV significantly reduces both SQV and DLV concentrations.
Goebel et al. [101] (2010)	RCT	EU	Tot: 208 -G1(TPV/r 1250/100 mg): 58 -G2(TPV/r 750/100 mg): 63 -G3(TPV/r 250/200 mg): 87	-G1: 22.4 -G2: 14.3 -G3: 12.6	18–75	-G1: 43 -G2: 39 -G3: 39	TPV/r and existing HAART regimen (AZT/3TC/ABC; AZT/3TC/EFV; AZT/3TC/NVP; d4T/3TC/EFV; d4T/3TC/NVP; d4T//EFV; d4T/ddI/NVP)	22 days for most HAART regimens 21 days EFV	PK effects of TPV/r co-administered with HAART; safety and efficacy of HAART	Co-administration of TPV/r is safe and effective, with a low potential for clinically significant DDIs, when integrated into HAART regimens containing NVP, EFV, 3TC, d4T, or ddI.
Sekar et al. [102] (2009)	RCT	USA	19	26	33–56	43	NVP, DRV/r	14 days	PK and DDIs between DRV/r and NVP	No significant DDIs between DRV/r and NVP. No dosage adjustments are necessary.
Wenning A. et. [69] (2008, study B)	Study B: RCT with placebo	USA	Study B: 104	4	22–68	NA	RAL, TDF, 3TC	NA	PK and DDIs between RAL and TDF	The co-administration of RAL and TDF does not significantly affect the PK of either drug. RAL and TDF can be administered together without any dose adjustments.
Vogler et al. [103] (2010)	PK study	USA	Tot: 32 -G1(LPV/r, EE, NGMN): 8 -G2(EE, NGMN): 24	-G1: 100 -G2: 100	18–49	-G1: 28 -G2: 31	LPV/r and EE and NGMN administered transdermally	4 weeks	PK and DDIs between LPV/r and EE and NGMN administered transdermally.	A significant difference in patch EE and NGMN concentrations has been observed in LPV/r-treated subjects. These patients show low levels of EE and high levels of NGMN. while maintaining contraceptive efficacy.
DuBois et al. [104] (2015)	PK study	USA	Tot: 27 -G1 (NETA+ RTV+ATV): 10 -G2 (NETA): 17	-G1: 100 -G2: 100	-G1: 18–44 -G2: 18–44	-G1: 37.5 -G2: 37.9	NETA with RTV-boosted ATV therapy	21 days	PK of NETA	Progestin-only contraceptives show higher drug exposure when co-administered with RTV-enhanced ATV regimens.
Luque et al. [105] (2015)	PK study	USA	25	100	15–47	32	DMPA, LPV/r	12 weeks	PK, DDIs and safety of LPV/r and DMPA	The simultaneous administration of LPV/r and DMPA determines a significant increase in DMPA exposure. Despite this, MPA is relatively well tolerated. The most side effects reported are mild or moderate irregular vaginal bleedings.
Rudek et al. [106] (2014)	PK study	USA	19	0	38–71	54	HAART, sunitinib	12 months	PK, safety and efficacy of HAART and sunitinib	Patients not in treatment with RTV tolerate sunitinib 50 mg/day and show toxicities comparable to those found in patients receiving a RTV-based HAART in treatment with sunitinib at the dosage of 37.5 mg/day. The recommended dosage for patients taking RTV is 37.5 mg daily after a 4-week on/2-week off regimen.
Ruxrungtham et al. [107] (2004)	Pk study	Asia	Tot: 25 -G1(RTV+ENF): 12 -G2(SQV/r+ENF): 13	-G1: 50 -G2: 54	-G1: 18–64 -G2: 18–64	NA	RTV, SQV/r, ENF	15 days	PK and DDIs between RTV, SQV/r and ENF	The co-administration of RTV or SQV/r does not significantly affect the PK of ENF. No serious ADRs or treatment discontinuations have been recorded.
Vieira et al. [108] (2014)	Prospective interventional PK study	USA	Tot: 45 -G1(LPV/r, AZT/3TC, ENG): 15 -G2(EFV, AZT/3TC, ENG): 15 -G3(ENG): 15	-G1: 100 -G2: 100 -G3: 100	18–40	-G1: 27.1 -G2: 34.5 -G3: 28.8	LPV/r, EFV, AZT, 3TC, ENG	6 months	PK and DDIs between LPV/r or EFV, and ENG-releasing implant	The simultaneous use of EFV reduces the bioavailability of ENG, potentially compromising its contraceptive effectiveness. In contrast, the combination of LPV/r enhances the bioavailability of ENG, indicating that this antiretroviral regimen does not negatively affect the efficacy of the ENG implant.
Yang et al. [109] (2022)	Retrospective cohort observational study.	Asia	Tot: 66 -G1(DTG/3TC/TDF): 20 -G2(DTG/ABT): 24 -G3 (BIC/TAF/FTC): 22	-G1: 35 -G2: 25 -G3: 63.6	-G1: 44–74 -G2: 45–85 -G3: 33–74	NA	INSTI-based regimens	6 month	Safety and efficacy between HAART and chemotherapy	The majority of AEs recorded during treatment are grade 1–2. Grade 3–4 AEs occur in 9.09% of patients, while no grade 5 AEs have been observed. HAART including DTG or BIC are effective choices for patients with HIV and colorectal cancer.
Neary et al. [110] (2019)	Pgx study	Africa	Tot: 57 -G1(ETON): 19 -G2 (ETON+ EFV): 19 -G3(ETON+ NVP): 19	-G1: 100 -G2: 100 -G3: 100	-G1: 24–30 -G2: 23–35 -G3: 28–35	-G1: 27 -G2: 29 -G3: 32	ETON, EFV or NVP	24 weeks	Influence of Pgx on the DDIs between ENG and EFV or NVP-based HAART.	Co-administration of EFV and ETON causes harmful DDIs regardless of the patient’s genetic background. These are worse in women with allelic variants of *CYP2B6* gene.
Perram et al. [111] (2019)	DDIs study	Australia	14	0	50–81	NA	Dabigatran, HAART	12 months	DDIs between dabigatran and HAART	No DDIs have been shown and no thromboembolic or hemorrhagic events have been observed.
Benesic et al. [112] (2004)	DDIs study	EU	Tot: 25 -G1 (HAART+FLUV): 12 -G2 (HAART+PRAV): 13	-G1: 25 -G2: 15	NA	NA	FLUV and PRAV with HAART	72 weeks	Effectiveness of FLUV and PRAV; DDIs between FLUV and PRAV with HAART	HAART therapy with FLUV and PRAV is effective in reducing total cholesterol and LDL levels. No effects on triglycerides, HDL or IDV plasma levels have been observed.

Abbreviations: ABC, Abacavir; ABT, Albuvirtide; ADR, Adverse Drug Reactions; ADV, Adefovir Dipivoxil; ATV, Atazanavir; AZT, Zidovudine; BIC, Bictegravir; BIC/FTC/TAF, Bictegravir/Emtricitabine/Tenofovir Alafenamide; d4T, Stavudine; ddI, Didanosine; DDIs, Drug–drug interactions; DLV, Delavirdine; DMPA, Depot Medroxyprogesterone Acetate; DRV/r, Darunavir/Ritonavir; DTG, Dolutegravir; EE, Ethinyl estradiol; EFV, Efavirenz; ENF, Enfuvirtide; ENG, Etonorgestrel; ETON, Etonogestrel Contraceptive Implant; EU, Europe; FLUV, Fluvastatin; HAART, Highly Active Anti-Retroviral Therapy; HDL, High-Density Lipoprotein; IDV, Indinavir; INSTIs, Integrase inhibitors; LDL, Low-Density Lipoprotein; LPV/r, Lopinavir-ritonavir; MPA, Medroxyprogesterone Acetate; NA, Not Available; NETA, Norethindrone; NGMN, Norelgestromin; NVP, Nevirapine; Pgx, Pharmacogenetics; PK, Pharmacokinetics; PRAV, Pravastatin; RAL, Raltegravir; RTV, Ritonavir; SQV, Saquinavir; SQV/NFV, Saquinavir/nelfinavir; SQV/r, Saquinavir/ritonavir; TDF, Tenofovir; TPV/r, Tipranavir/Ritonavir; 3TC, Lamivudine; USA, United State of America.

**Table 3:** Main characteristics and results of the studies, published between 2000 and 2024, that have investigated DDIs between HAART and co-administered drugs used for the treatment of co-infections (tuberculosis, malaria, HBV) in HIV mono-infected or co-infected patients. All drugs reported in these studies were orally administered.

References	Study Type	Country	Patients (n)	Sex (F%)	Range (Years)	Median (Years)	Type of Co-Infection	Drugs Involved	Follow-Up	Outcomes	Main Results and Conclusions
Atwine et al. [113] (2020)	RCT	Africa	Tot: 97-G1 (600 mg EFV+ 600 mg RIF): 33 -G2(1200 mg EFV+ 600 mg RIF): 31 -G3(1200 mg EFV+ 800 mg RIF): 33	-G1: 12.1 -G2: 29 -G3: 39.4	NA	-G1: 34.1 -G2: 33.4 -G3: 32.3	TB	RIF, EFV	28 weeks	PK parameters of EFV; RIF Cmax; treatment adherence and safety	Co-administration of a doubled dose of RIF with EFV in HIV/TB co-infected patients is generally well tolerated. RIF dose doubling is safe and effective for the treatment of TB and HAART.
De Kock et al. [114] (2014)	RCT	Africa	73	46.6	18–64	33	TB	PAS, EFV	12 weeks	PK of PAS; DDIs between PAS and EFV; tolerance and safety	PAS concentrations are lower in patients co-infected with HIV and TB, and the use of EFV with PAS may contribute to this reduction, although the exact mechanism of the drug interaction remains unclear. When HAART includes EFV, administering 4 g of PAS twice daily seems to be both effective and well-tolerated, whereas higher doses, such as 12 g once daily, show reduced effectiveness.
Lopez-Cortes et al. [115] (2002)	RCT	EU	24	14.3	22–58	37	TB	EFV, RIF	6 months	PK interactions between EFV and RIF	EFV daily dose of 800 mg in patients with HIV and TB may increase the efficacy of therapy when co-administered with RIF. RIF can be used with EFV without requiring dosage changes.
Zhang et al. [116] (2013)	RCT	Africa	Tot: 95 -G1(Children): 74 -G2(Adults): 21	-G1: 42.1 -G2: 18.9	NA	-G1: 21 months -G2: 36years	TB	LPV/r, RIF	1 month	Influence of RIF on PK of LPV/r	In children, LPV bioavailability decreases more with RTV than in adults. RIF increases LPV and RTV clearance more in adults compared to children.
Podany et al. [117] (2021)	RCT	USA	78	78	13–66	40	TB	NVP, EFV, RPT, INH	4 weeks	PK and efficacy and safety of RPT and INH vs. the standard INH regimes in association with HAART	High-dose RPT modestly decreases EFV clearance. Therapeutic targets are achieved without EFV dose adjustments.
Wallender et al. [118] (2018)	RCT with placebo	Africa	83	100	16–43	NA	Malaria	DHA-PQ, EFV	40 weeks of gestation	PK and DDIs between EFV and DHA-PQ	In HIV+ pregnant women taking EFV, daily low-dose DHA-PQ offers better protection against parasitemia and shows lower toxicity risks compared to monthly dosing schedules.
Scarsi et al. [119] (2023)	RCT	Africa, Asia and USA	Tot: 118 -G1(LNG 1.5 mg + DGT): 32 -G2(LNG1.5 mg +EFV): 17 -G3(LNG 3 mg+EFV): 35 -G4(LNG 3 mg+RIF): 34	-G1: 62.5 -G2: 47.1 -G3: 60 -G4: 50	-G1: 18–55 -G2: 20–58 -G3: 22–54 -G4: 19–60	-G1: 34 -G2: 42 -G3: 36 -G4: 25	TB	LNG, EFV and RIF	12 months	PK of LNG and EFV or RIF	Doubling the dose of LNG-based emergency contraceptive increases LNG exposure in the first 8 h in subjects receiving EFV-based ART or RIF -based TB therapy.
Byakika-Kibwika et al. [120] (2012)	PK study	Africa	Tot: 58 -G1(AL+ EFV): 30 -G2 (AL+NVP): 28	-G1: 63 -G2: 96	NA	-G1: 38 -G2: 33	Malaria	AL, EFV, NVP	10 weeks	DDIs between AL and EFV or NVP	Co-administration of AL with EFV or NVP leads to a significant reduction in the exposure levels of artemether and its active metabolite, dihydroartemisinin. Additionally, lumefantrine exposure is significantly decreased when taken with EFV, while the reduction with NVP is not statistically significant.
Meyers et al. [121] (2019)	PK study	Africa	Tot: 26 -G1(2–5 years): 12 -G2 (6–12 years): 14	G1: 41.6 -G2: 50	-G1: 2 -5 -G2: 6–12	-G1: 3 -G2: 8	TB	RAL, RIF, EFV, LPV/r, NRTIs	12 weeks following the cessation of RAL	PK and safety of RAL and RIF	***Administration of 12 mg/kg RAL chewable twice daily safely achieves PK targets in HIV/TB children***
Ren et al. [122] (2008)	PK study	Africa	Tot: 30 -G1(RIF and LPV/r): 15 -G2(LPV/r): 15	-G1: 53 -G2: 67	NA	-G1: 16 -G2: 29	TB	RIF, LPV/r	20 weeks	PK of LPV/r and RIF	***The reduction in LPV Cmin caused by RIF can be attenuated by adding RTV to LPV. An adjusted dose of LPV/r results in adequate Cmin of LPV in most HIV-infected children co-administered with RIF.***
Ren et al. [123] (2009)	Observational PK study.	Africa	15	NA	NA	6.3	TB	EFV, RIF	78 weeks	PK of RIF and EFV	Co-administration of RIF and EFV does not lead to a significant decrease in estimated Cmin levels of EFV in children co-infected with HIV and TB. Therefore, no significant DDIs were detected between RIF and EFV.
Scarsi et al. [124] (2014)	Observational PK study.	Africa	21	71.4	NA	39.7	Malaria	AZT, NVP, 3TC, AS/AQ	6 days	DDIs between AS/AQ and HAART; PK of HAART and AQ (DEAQ metabolite)	Patients on AZT-based ART show reduced exposure to AQ and its active metabolite DEAQ, with possible reduction of AS/AQ effectiveness.
Sevene et al. [125] (2019)	PK study	Africa	Tot: 221 -G1 (EFV+ DPQ): 160 -G2 (NVP+DPQ): 61	-G1: 66.5 -G2: 75.4	15–65	-G1: 38.6 -G2: 47	Malaria	DPQ, EFV, NVP	63 days	Efficacy and safety of DPQ in patients on EFV- or NVP-based HAART; PK interactions between HAART and DPQ	Treatment with DPQ is highly effective and safe for adult HIV+ patients receiving EFV or NVP-based HAART with uncomplicated malaria, despite known PK interactions. A prolongation of the QTc has been observed, with spontaneous resolution within 14 days.
Tanuma et al. [126] (2013)	PK study	Asia	16	0	23–60	36	TB	RFB, LPV/r	2 years	PK of two RFB regimens (300 mg/day RFB alone versus 150 mg every other day) in combination with LPV/r	A dose of RFB of 150 mg taken every other day, combined with a LPV/r-based HAART, produces a similar AUC as that observed with a standard dose of 300 mg daily. The use of the reduced dose results in a lower exposure to RFB.
van der Laan et al. [127] (2021)	Prospective PK study	Africa	54	53.7	0–15	5.7	TB	PZA, TRD, EMB, ETA, INH, 3TC, ABC	52 weeks	PK and DDIs between ABC and 3TC	The TB treatment regimen including PZA, EMB, ETA, INH, TRD, do not have a significant impact on the PK of 3TC or ABC.
Narita et al. [128] (2000)	DDI study	USA	25	36	27–65	36	TB	RFB with PIs (IDV or NFV) or NNRTI	13 months	DDIs between RFB and PIs or NNRTI	The combination of RFB and PI is effective in HIV/TB co-infection. RFB retains its bactericidal activity, while PI maintains viral suppression without exacerbating side effects.
Moh et al. [129] (2005)	DDI study	Africa	498	72	29–41	34	Hepatitis B	AZT, 3TC, EFV, TMP-SMX	6 months.	DDIs between AZT-based HAART and TMP-SMX; incidence of hematological disorders.	Co-administration of TMP-SMX and AZT leads to a significant increase in grade 3–4 neutropenia, which resolves after TMP-SMX discontinuation.
Liou et al. [130] (2021)	Observational prospective study	Asia	48	2.1	37–51	41.0	LTBI	INH, RPT, BIC/FTC/TAF	52 weeks	Safety and tolerability of RPT-INH treatment in HIV+ patients with LTBI also receiving BIC/FTC/TAF therapy.	Treatment with RPT-INH in combination with BIC/FTC/TAF is well tolerated. Therefore, it has significant effects on BIC trough plasma concentrations.
Martinson et al. [131] (2009)	Retrospective cohort study	Africa	1132	50.3	NA	6.3	TB	RFB, RIF, INH, PZA, EMB, LPV/r, EFV, RAL 3TC, d4T, AZT, NVP	12 weeks	HAART efficacy in reducing the incidence of TB	In HIV+ children, HAART is associated with a 70% reduction in the risk of childhood TB incidence.

Abbreviations: ABC, Abacavir; AL, Artemether-Lumefantrine; AQ, Amodiaquine; AS/AQ, Artesunate/Amodiaquine; AUC, Area Under The Curve; AZT, Zidovudine; BIC, Bictegravir; BIC/FTC/TAF, Bictegravir/Emtricitabine/Tenofovir Alafenamide; Cmax, Maximum Plasma Concentration; Cmin, Minimum Plasma Concentration; d4T, Stavudine; DDIs, Drug–Drug Interactions; DEAQ, Desethylamodiaquine; DHA-PQ, Dihydroartemisinin–Piperaquine; DPQ, Dihydroartemisinin–Piperaquine; EFV, Efavirenz; EMB, Ethambutol; ETA, Ethionamide; EU, Europe; G, Group; HAART, Highly Active Anti-Retroviral Therapy; HIV, human immunodeficiency virus; IDV, Indinavir; INH, Isoniazid; LNG, Levonorgestrel; LPV, Lopinavir; LPV/r, Lopinavir-ritonavir; LTBI, Latent Tuberculosis Infection; NA, Not Available; NFV, Nelfinavir; NNRTI, Non-Nucleoside Reverse Transcriptase Inhibitor; NRTIs, Nucleoside Reverse Transcriptase Inhibitors; NVP, Nevirapine; PAS, Para -Aminosalicylic Acid; PIs, Protease Inhibitors; PK, Pharmacokinetics; PQ, Piperaquine; PZA, Pyrazinamide; RAL, Raltegravir; RFB, Rifabutin; RIF, Rifampicin; RPT, Rifapentine; RTV, Ritonavir; TB, Tuberculosis; TMP-SMX, Co-Trimoxazole; TRD, Terizidone; USA, United State of America; 3TC, Lamivudine.

**Table 4:** Studies, published between 2000 and 2024, investigating DDIs between HAART and co-administered drugs used for the treatment of HCV in healthy and HIV/HCV co-infected patients. All drugs reported in these studies were orally administered.

References (Years)	Study Type	Country	Patients (n)	Sex (F%)	Median Age (Years)	Age Range (Years)	Drugs Involved	Follow-Up	Outcomes	Main Results and Conclusion
**HIV/HCV coinfected patients**										
Poizot-Martin et al. [161] (2003)	Retrospective and prospective study	EU	Tot: 62 -G1(IFN+RBV): 42 -G2(PegIFN+RBV): 20	-G1: 28.1 -G2: 20	-G1: 36 -G2: 37	-G1: 34–40 -G2: 33–40	IFN alpha-2b (peg or not) plus RBV and HAART	At least 6 and up to 12 months	Therapeutic success	HAART with PI is associated with HCV therapeutic failure (*p* = 0.049).
Carrat et al. [139] (2004)	RCT	EU	Tot: 412 HAART with -G1(pegIFN alfa-2b+RBV): 205 -G2(IFNalfa-2b+RBV): 207	-G1: 23 -G2: 29	NA	NA	PegINF alfa-2b or IFN alfa-2b plus RBV with HAART	72 weeks	Virologic efficacy and safety	Predictors of response included no treatment with PIs. Serious AEs were much more frequent than reported among HIV-negative patients. Mitochondrial toxicity was particularly frequent in patients receiving DDI.
Chung et al. [140] (2004)	RCT	USA	Tot: 133 HAART with -G1(pegIFN alfa-2b+RBV): 66 -G2(IFNalfa-2b+RBV): 67	-G1: 21 -G2: 15	-G1: 45 -G2: 44	NA	PegINF alfa-2b or IFN alfa-2b plus RBV with HAART	24 weeks	Virologic efficacy and safety	In multivariate analysis, the use or non-use of any ARV therapy and the use or non-use of PIs are not predictive of a SVR. Only one episode of pancreatitis occurred that required hospitalization and discontinuation of treatment. The subject was also receiving didanosine.
Torriani et al. [141] (2004)	RCT with placebo	USA	Tot: 860 HAART with -G1(PegIFN alfa-2b+RBV): 289 -G2(PegIFN alfa-2b+placebo): 286 -G3(IFN+RBV): 285	-G1: 20 -G2: 18 -G3: 19	NA	NA	PegINF alfa-2b or IFN alfa-2b plus RBV with HAART	72 weeks	SVR and safety	The use or non-use of any ARV therapy is not predictive of a SVR.
Gutierrez-Valencia et al. [149] (2014)	PK study	EU	Tot: 14	0	47	42–56	TVR + PegIFN-alpha and RBV with RTV-boosted HIV-PIs	NA	PK	Co-administration of TVR and unboosted ATV results in increased exposure of both drugs compared with co-administration with RTV.
Venuto et al. [150] (2020)	PK study	USA	Tot: 11	18	44.8	NA	OBV/PTV/r + DSV and RAL	35 days	PK	The plasma concentrations of RAL of co-infected patients is, in most cases, lower after initiation of HCV therapy; however, the confidence intervals are wide.
MacBrayne et al. [162] (2018)	NA	USA	Tot: 37 TDF with: -G1 (SOF+RBV): 15 -G2 (SOF+LPV): 22	0	NA	NA	SOF/RVB, SOF/LPV and TDF	12 weeks	Measurement of tenofovir levels in plasma, tenofovir diphosphate in dried blood spots, and tenofovir diphosphate in PBMCs	In patients taking SOF/RVB, tenofovir diphosphate is 4.3-fold higher (*p* = 0.0001) in dried blood spots and 2.3-fold higher (*p* = 0.03) in PBMCs versus study entry. In patients taking SOF/LPV, tenofovir diphosphate is 17.8-fold higher (*p* < 0.0001) in dried blood spots versus study entry. Tenofovir plasma concentrations are 2.1-fold higher (*p* = 0.0005).
Rodriguez-Torres et al. [163] (2015)	NA	USA	Tot: 38 -G1 (SOF+EFV/FTC/TDF): 12 -G2 (SOF+EFV/ZDV/3TC): 4 -G3 (SOF+ ATV/r/FTC/TDF): 8 -G4 (SOF+DRV/r/FTC/TDF): 7 -G5 (SOF+FTC/TDF/RAL): 7	-G1: 16.7 -G2: 0 -G3: 12.5 -G4: 14.3 -G5: 14.3	NA	-G1: 41–70 -G2: 51–64 -G3: 37–53 -G4: 42–54 -G5: 31–58	SOF and EFV, 3TC, TDF, ZDV, FTC, ATV, RTV, DRV, RAL	NA	Efficacy and safety	No clinically significant DDIs were observed between SOF and the anti-HIV evaluated.
**Healthy volunteers**										
Hulskotte et al. [142] (2013)	RCT	EU	Tot: 39 -G1 (BOC+ATV-r): 13 -G2 (BOC+LPV-r): 13 -G3 (BOC+DRV-r): 13	-G1: 31 -G2: 38 -G3: 38	NA	NA	BOC, RTV-boosted PIs	31 days	PK and safety	BOC reduces the exposure of all PI/r and itself. Treatments are well tolerated with no unexpected AEs.
Khatri et al. A [151] (2016)	PK studies	USA	Tot: 144 -G1(3D+ DRVb or DRV+rc): 36 -G2(3D+DRV+rd): 24 -G3(3D+ATVb orATV+rd): 48 -G4(3D+LPV/rd): 24 -G5(3D+LPV/rc): 12	-G1: 6 -G2: 29 -G3: 33 -G4: 8 -G5: 25	NA	33–38	OBV, PTV/r, and DSV (3D regimen) with ATV, DRV, LPV/r	28 days	PK, safety and tolerability	Evening administration of ATV/r or LPV/r with the 3D regimen is not recommended, given the higher exposure to PTV and/or ritonavir found. An increase in total bilirubin levels is identified in the ATV arm.
Feng et al. A [152] (2019)	PK study	EU	Tot: 79 -G1(GZR+RTV): 10 -G2(GZR+ATV/r: 13 -G3(GZR+LPV/r): 13 -G4(GZR+DRV/r): 13 -G5(EBR+ATV/r): 10 -G6(EBR+LPV/r): 10 -G7(EBR+DRV/r): 10	-G1: 0 -G2: 31 -G3: 46 -G4: 31 -G5: 40 -G6: 40 -G7: 40	-G1: 30.7 -G2: 40 -G3: 37 -G4: 44 -G5: 31 -G6: 35 -G7: 34	-G1: 24–44 -G2: 25–49 -G3: 39–47 -G4: 28–55 -G5: 20–48 -G6: 21–52 -G7: 23–49	EBR and GZR with RTV and RTV-boosted HIV-PIs	NA	PK and safety	Coadministration of EBR-GZR with PI/r is contraindicated, due to an increase in GZR exposure. HIV treatment regimens without RTV-boosted HIV-PIs should be considered.
Kosloski et al. [159] (2020)	PK-DDI study	USA	Tot: 165 GLE and PIB with: -G1(EVG/c/FTC/TAF): 24 -G2(ABC/DTG/3TC): 24 -G3(RAL): 12 -G4(RPV): 24 -G5(DRV/r): 24 -G6(ATV/r): 24 -G7(LPV/r): 21) -G8(EFV/FTC/TDF): 12	-G1: 8 -G2: 4 -G3: 8 -G4: 38 -G5: 25 -G6: 42 -G7: 24 -G8: 0	NA	NA	GLE and PIB with ARV regimens	NA	Potential DDI and systemic drug exposure	The AUC of GLE is increased more than 4-fold when taken together with RTV-boosted HIV-PIs, while PIB concentrations are not affected. In contrast, GLE and PIB exposure may be significantly reduced by concomitant intake of EFV. Increases in alanine transaminase have occurred in combination with ATV/r.
Bifano et al. B [153] (2013)	PK studies	USA	Tot: 52 -G1(DCV+ATV/r): 14 -G2(DCV+EFV): 17 -G3(DCV+TDF): 21	-G1: 21 -G2: 9 -G3: 5	NA	-G1: 8–43 -G2: 18–48 -G3: 20–49	DCV and ATV/r, EFV and TDF.	22 days	PK and safety	Dose adjustment of DCV to 30 mg once daily with ATV/r and 90 mg once daily with EFV should normalize AUC to target exposure. DCV is well tolerated in combination with all three ARVs studied.
Sabo et al. [154] (2014)	PK studies	USA and EU	Tot: 45 -G1(DRV/r+FDV): 14 -G2(FDV + EFV): 15 -G3(FalFDVdaprevir + TDF): 16	-G1: 14.3 -G2: 40 -G3: 18.7	-G1: 42.0 -G2: 31 -G3: 37	-G1: 20–55 -G2: 20–53 -G3: 25–54	FDV with DRV/r, EFV, tenofovir	22 days	PK and safety	Co-administration of EFV and FDV results in a relevant decrease in FDV exposure. This decrease could be managed by using the higher dose among the 2 doses of FDV tested in the phase 3 studies.
Mogalian et al. [143] (2018)	RCT	USA	Tot: 237 SOF/VEL With or Without -G1(EFV/FTC/TDF): 30 -G2(FTC/RPV/TDF): 24 -G3(DTG): 24 -G4(FTC/TDF+RAL): 30 -G5(EVG/c/FTC/TAF): 23 -G6(EVG/c/FTC/TDF): 24 -G7 (ATV+RTV+FTC/TDF: 24 -G8 (DRV+RTV+FTC/TDF: 30 -G9 (LPV/RTV+FTC/TDF: 23	-G1: 40 -G2: 42 -G3: 42 -G4: 40 -G5: 37 -G6: 46 -G7: 29 -G8: 45 -G9: 50	NA	-G1: 19–45 -G2: 22–45 -G3: 22–44 -G4: 21–45 -G5: 20–44 -G6: 21–45 -G7: 26–45 -G8: 20–45 -G9: 22–44	SOF/VEL with multiple ARV regimens	NA	PK, safety and tolerability	Use of SOF/VEL with EFV-containing regimens is not recommended due to an approximate 50% reduction in VEL exposure.
Ashby et al. [155] (2011)	PK study	EU	Tot: 14	29	NA	NA	RVB, RAL	35 days	PK and safety	Cmax decreases and Tmax increases when RBV is taken together with RAL. However, this is unlikely to have clinical significance or impact on the antiviral effects of RBV.
Joseph et al. [156] (2015)	PK study	USA	Tot: 24	50	NA	NA	FDV, RAL	NA	PK and safety	Compared with RAL alone, co-administration with FDV results in a 2.7-fold and 2.5-fold increase in the mean AUC and Cmax of RAL, respectively. The incidence of AEs is higher during combination treatment than during treatment with RAL alone. No serious AEs are reported.
Khatri et al. B [157] (2016)	PK studies	USA	Tot: 66 -G1(3D+FTC+TDF): 18 -G2(3D+RPV): 20 -G3(2D+EFV/TDF/FTC): 16 -G4(3D+RAL): 12	-G1: 0 -G2: 25 -G3: 6.3 -G4: 33.3	NA	NA	OBV, PTV/r, and DSV (3D or 2D regimen)with RAL, TDF, FTC, EFV and RPV	21 days	PK and safety	Exposure to RAL increased when the drug is administered with the 3D regimen. Increased exposure to RPV may raise the potential for ADRs when RPV is administered with the 3D regimen. Concomitant use of the 2D regimen and EFV/TDF/FTC has been discontinued due to intolerability and adverse events.
Feng et al. [158] (2019)	PK study	USA	Tot: 36 -G1(EBR+TDF): 10 -G2(GZR+TDF): 12 -G3(EBR-GZR+TDF): 14	-G1: 40 -G2: 25 -G3: 43	NA	-G1: 21–53 -G2: 22–50 -G3: 20–55	EBR-GZR and TDF	NA	PK and safety	EBR and/or GZR co-administration with TDF did not result in significant alterations in TDF exposure and is generally well tolerated, with no deaths or serious AEs.
Ankrom et al. [160] (2019)	PK- DDI studies	USA	Tot: 26 -G1:(EBR+GZR): 12 -G2(LDV+SOF): 14	-G1: 58.3 -G2: 14.3	-G1: 29 -G2: 36	-G1: 25–55 -G2: 25–60	DOR, EBR/GZR, SOF /LDV	NA	PK	No significant PK interactions have beeen observed when DOR is coadministered with anti HCV EBR/GZR or LDV/SOF. DOR can be taken alongside HCV regimens without requiring dose adjustments.
Custodio et al. [144] (2017)	RCT	USA	Tot: 42	28.6	NA	18–45	SOF/VEL, RPV/F/TAF	30 days after discontinuation of study drug	PK and safety	The Cmax and AUC of tenofovir, the main metabolite of TAF, increased by 62% and 75%, respectively. However, the resulting absolute tenofovir exposures were not considered clinically relevant.
De Kanter et al. [145] (2013)	RCT	EU	Tot: 24	50	NA	20–55	BOC, RAL	NA	DDI between BOC and RAL	RAL can be recommended for combined HIV/HCV treatment including BOC.
Feng et al. [146] (2019)	RCT	USA	Tot: 34 -G1(EBR+RAL): 10 -G2(GZR+RAL): 12 -G3(EBR-GZR+DTG): 12	-G1: 40 -G2: 42 -G3: 33	NA	-G1: 20–47 -G2: 21–51 -G3: 27–52	EBR-GZR and DTG or RAL	NA	PK and safety	No clinically significant DDIs were found between EBR or GZR with RAL or DTG. The treatments are well tolerated.
Johnson et al. [147] (2014)	RCT	USA	Tot: 32 -G1(DTG+BOC): 16 -G2(DTG+TPV): 16	-G1: 38 -G2: 44	NA	NA	BOC and TPV, DTG	14 days	PK	DTG can be given concurrently with BOC or TPV to HIV/HCV coinfected patients without requiring dose adjustments.
Kakuda et al. [148] (2014)	RCT	USA and EU	Tot: 33 -G1(etravirine with/without TVR): 17 -G2(rilpivirine with/without TVR): 16	-G1: 64.7 -G2: 50	-G1: 48.0 -G2: 41.5	-G1: 18–54 -G2: 23–52	TVR, RPV or etravirine	19 days	PK	TVR does not affect the PK of etravirine but increases the Cmin, Cmax, and AUC of rilpivirine by 93%, 49%, and 78%, respectively. This interaction does not appear to be clinically relevant, and dose adjustment is not necessary when co-administered.

Abbreviations: ABC, Abacavir; AEs, Adverse events; ADRs, Adverse Drug Reactions ARV, Antiretroviral; ATV, Atazanavir; ATV/r, Atazanavir/ritonavir; AUC, Area Under the Curve; BOC, Boceprevir; Cmax, Maximum plasma concentration; Cmin, Minimum plasma concentration; DCV, Daclatasvir; DDI, Didanosine; DRV, Darunavir; DRV/r, Darunavir/ritonavir; DSV, Dasabuvir; DTG, Dolutegravir; EBR, Elbasvir; EFV, Efavirenz; EVG, Elvitegravir; EU, Europe; FDV, Faldaprevir; FTC, Emtricitabine; G(n), Group; GLE, Glecaprevir; GZR, Grazoprevir; HAART, Highly Active Antiretroviral Therapy; HCV, Hepatitis C Virus; HIV, Human Immunodeficiency Virus; IFN, Interferon; PegIFN, pegylated interferon; LDV, ledipasvir; LPV/r, Lopinavir/ritonavir; LVP, Ledipasvir; OBV, Ombitasvir; PK, Pharmacokinetis; PTV/r, Paritaprevir/ritonavir; PIB, Pibrentasvir; PIs, Protease Inhibitors; RAL, Raltegravir; Rb, Once-daily dosing with morning administration; rc, Twice-daily dosing with morning and evening administration; RBV, Ribavirin; RCT, Randomized Controlled Trial; rd, Once-daily dosing with evening administration; RPV, RPV/F/TAF, Rilpivirine; rilpivirine/emtricitabine/tenofovir alafenamide; RTV, ritonavir; SOF/LPV, Sofosbuvir/ledipasvir; SOF/RBV, Sofosbuvir/ribavirin; SOF/VEL, Sofosbuvir/velpatasvir; SVR, Sustained Virologic Response; TAF, Tenofovir alafenamide; TDF, Tenofovir Disoproxil Fumarate; Tmax, Time to peak drug concentration; TVR, Telaprevir; USA, United States of America; ZDV, zidovudine; 2D regimen, paritaprevir/ritonavir and dasabuvir; 3D regimen, Ombitasvir + Paritaprevir/ritonavir + Dasabuvir; 3TC, Lamivudine.

## Data Availability

The data that support the findings of this study are openly available.
